# Identification of an RNA sponge that controls the RoxS riboregulator of central metabolism in *Bacillus subtilis*

**DOI:** 10.1093/nar/gkab444

**Published:** 2021-06-07

**Authors:** Sylvain Durand, Adam Callan-Sidat, Josie McKeown, Stephen Li, Gergana Kostova, Juan R Hernandez-Fernaud, Mohammad Tauqeer Alam, Andrew Millard, Delphine Allouche, Chrystala Constantinidou, Ciarán Condon, Emma L Denham

**Affiliations:** UMR8261, CNRS, Université de Paris, Institut de Biologie Physico-Chimique, 13 rue Pierre et Marie Curie, 75005 Paris, France; Division of Biomedical Sciences, Warwick Medical School, University of Warwick, Gibbet Hill Road, Coventry, UK; Division of Biomedical Sciences, Warwick Medical School, University of Warwick, Gibbet Hill Road, Coventry, UK; Division of Biomedical Sciences, Warwick Medical School, University of Warwick, Gibbet Hill Road, Coventry, UK; UMR8261, CNRS, Université de Paris, Institut de Biologie Physico-Chimique, 13 rue Pierre et Marie Curie, 75005 Paris, France; School of Life Sciences, Proteomics Research Technology Platform, University of Warwick, Gibbet Hill Road, Coventry, UK; Division of Biomedical Sciences, Warwick Medical School, University of Warwick, Gibbet Hill Road, Coventry, UK; Division of Biomedical Sciences, Warwick Medical School, University of Warwick, Gibbet Hill Road, Coventry, UK; UMR8261, CNRS, Université de Paris, Institut de Biologie Physico-Chimique, 13 rue Pierre et Marie Curie, 75005 Paris, France; Division of Biomedical Sciences, Warwick Medical School, University of Warwick, Gibbet Hill Road, Coventry, UK; UMR8261, CNRS, Université de Paris, Institut de Biologie Physico-Chimique, 13 rue Pierre et Marie Curie, 75005 Paris, France; Division of Biomedical Sciences, Warwick Medical School, University of Warwick, Gibbet Hill Road, Coventry, UK

## Abstract

sRNAs are a taxonomically-restricted but transcriptomically-abundant class of post-transcriptional regulators. While of major importance for adaption to the environment, we currently lack global-scale methodology enabling target identification, especially in species without known RNA hub proteins (e.g. Hfq). Using psoralen RNA cross-linking and Illumina-sequencing we identify RNA–RNA interacting pairs *in vivo* in *Bacillus subtilis*, resolving previously well-described interactants. Although sRNA–sRNA pairings are rare (compared with sRNA–mRNA), we identify a robust example involving the conserved sRNA RoxS and an unstudied sRNA RosA (Regulator of sRNA A). We show RosA to be the first confirmed RNA sponge described in a Gram-positive bacterium. RosA interacts with at least two sRNAs, RoxS and FsrA. The RosA/RoxS interaction not only affects the levels of RoxS but also its processing and regulatory activity. We also found that the transcription of RosA is repressed by CcpA, the key regulator of carbon-metabolism in *B. subtilis*. Since RoxS is already known to be transcriptionally controlled by malate via the transcriptional repressor Rex, its post-transcriptional regulation by CcpA *via* RosA places RoxS in a key position to control central metabolism in response to varying carbon sources.

## INTRODUCTION

To adapt to changing environments and survive exposure to harsh conditions, organisms have evolved complicated metabolic and genetic regulatory networks to ensure that a homeostatic balance is maintained (1,2). At the RNA synthesis level, gene expression can be modulated through combinations of transcription factors controlling genes required for growth and survival under specific conditions (3–5). At the post-transcriptional level, small regulatory RNAs (sRNAs) act to temper gene expression by short imperfect base pairing with their mRNA targets, altering the level of protein production by increasing or decreasing access to the ribosome-binding site, or by facilitating or blocking the access to the mRNA by ribonucleases (RNases) (6,7). Most regulatory RNAs are independently expressed under the control of specific transcription factors. However, more recently, it has been shown that sRNAs can also be produced by processing RNAs that have other functions in the cell, such as tRNAs (8) and mRNAs (9).

Regulation by RNA is an important mechanism for fine-tuning gene expression in the Gram-positive model bacterium *Bacillus subtilis*, recently reviewed in (10). Over 150 potential sRNAs have been identified in *B. subtilis* and shown to be expressed in a condition-dependent fashion (11–13). To date the roles of very few of these putative sRNAs have been determined. Where targets have been identified, they have been shown to play key roles in stress adaptation. *B. subtilis* notably expresses three sRNAs with C-rich regions (CRRs) with similar predicted secondary structure; RoxS/S415 (Related to oxidative stress) (14), FsrA/S512 (Fur regulated small RNA) (15) and CsfG/S547 (Controlled by sigma-F and sigma-G) (16), (S numbers relate to transcriptionally active segments identified by Nicolas *et al.* (12)). The RoxS sRNA is one of the best characterised sRNAs in Gram-positive bacteria (14,17,18) and is conserved among Bacilli and Staphylococci, where it is named RsaE (19,20). RoxS has been shown to be upregulated in response to nitric oxide (NO) in *B. subtilis* and *S. aureus*, by the two-component system ResDE, and its homolog SsrAB, respectively (14). RoxS expression is also activated when malate is supplied as a carbon source (17). This control is mediated by the transcription factor Rex, that senses the NAD/NADH ratio of the cell. Indeed, this ratio is perturbed by the conversion of malate to pyruvate by the three malate dehydrogenases of *B. subtilis* that reduce NAD^+^ to NADH, and by its cycling through the TCA pathway. It has been proposed that one role of RoxS is to re-equilibrate the NAD/NADH ratio of the cell by inhibiting the expression of enzymes that produce NADH. FsrA is regulated by the transcription factor Fur and acts as part of the iron-sparing response (15). Fur down-regulates mRNAs whose protein products contain iron as part of their structures, but are not essential for growth, therefore ensuring iron availability for essential iron-containing proteins (15,21). Interestingly, both RoxS and FsrA down-regulate expression of several common genes encoding enzymes of the TCA cycle that produce NADH. CsfG is highly expressed during sporulation, anaerobic growth and after glucose exhaustion (12). During sporulation, expression of this sRNA is controlled by the sigma factors F and G, which are restricted to the forespore (16). However, to date no mRNA target or physiological role for CsfG has been identified. Durand *et al.* have hypothesised that its similar sequence motifs and structure to RoxS and FsrA suggests these three sRNAs may have overlapping targets and play similar roles under different growth conditions (14).

The lack of well resolved pathways through which sRNAs act in no small part reflects the difficulty of global scale target identification, this being more acute in some bacteria than others. In many enterobacteria, such as *Escherichia coli* and *Salmonella typhimurium*, the RNA chaperones Hfq and ProQ play key roles as mediators of sRNA–mRNA interactions and have greatly enabled the identification of mRNA targets through pull-down studies (22–25). ProQ is generally absent form Gram-positive bacteria, and although Hfq is present, it does not appear to play a global role in RNA-mediated regulation of gene expression (26,27). Hfq-dependent regulation by only one sRNA in *Listeria* and a handful in *Clostridium* are the only known exceptions (28,29). It is therefore generally accepted that sRNA regulation in the Firmicutes either depends on different RNA chaperones or can occur in the absence of any protein factors. A number of groups have used *in vivo* RNA cross-linking with the psoralen AMT, followed by ligation to form chimeras and RNA-seq to identify RNA–RNA interactions (30–33). Here we employed LIGR-seq (30) to identify sRNA targets in *B. subtilis*. The method was validated by identifying many known members of the FsrA and RoxS regulons and several new targets. We also identified a new regulatory RNA, S345, that interacts with both FsrA and RoxS. These interactions are found in independent samples and across multiple conditions. Given the possibility of a novel associated regulatory mechanism, and the rarity of well-characterised bacterial sRNA–sRNA interactions, we mechanistically dissect S345 and its interactants. We show that S345 not only functions as an RNA sponge for RoxS, but also affects its processing and degradation. We rename this sRNA RosA (for Regulator of sRNA A). We show that the transcription of RosA is under the control of the carbon catabolite control protein A (CcpA), linking the action of RoxS to the carbon source availability in *B. subtilis*.

## MATERIALS AND METHODS

### Media and growth conditions

Selection for transformations was performed on Lysogeny Broth (LB) at 37°C supplemented with required antibiotics. For *E. coli* these were ampicillin (100 μg ml^–1^) or chloramphenicol (10 μg ml^–1^) and for *B. subtilis*, phleomycin (4 μg ml^–1^), kanamycin (20 μg ml^–1^), tetracycline (5 μg ml^–1^), chloramphenicol (5 μg ml^–1^), erythromycin (2 μg ml^–1^), spectinomycin (100 μg ml^–1^) or combinations of the above. Growth experiments were performed in LB, M9 medium supplemented with glucose at a final concentration of 0.3% (12) or MD medium (34) supplemented with arabinose or malate at a final concentration of 1%.

### Bacterial strain construction

All *E. coli* and *B. subtilis* strains and plasmids used in this study are listed in Supplementary Table SI. Primer sequences can be found in Supplementary Table SII. *E. coli* DH5α and TG1 were used for all cloning procedures. *B. subtilis* strains were derived from the *B. subtilis* wild-type (WT) strain 168 *trp^+^* (W168). The isogenic deletion mutants were constructed according to the method described by Tanaka *et al.* (35) without pop-out of the deletion cassette. Transfer of genetic mutations between strains was achieved by transformation of genomic DNA extracted from the relevant strain. Reintroduction of sRNAs under the control of their native promoters was achieved by Gibson Assembly into pRMC that integrates into the *amyE* locus (36). Primer annealing sites were chosen to include the native promoter mapped in Nicolas *et al.* (12). The sequence of the cloned sRNAs was subsequently confirmed by sequencing and transformed into *B. subtilis* (plasmid pRMC+P_native_-sRNA). Integration into the *amyE* locus was confirmed by an iodine halo assay by replica plating transformation plates onto starch plates. The RosA promoter fusion was constructed at the native genomic locus by integration of the pBSBII plasmid (37). Combinatorial strains were constructed in the genetic background of the same promoter fusion strain by transformation of genomic DNA of the respective strain and selection on the appropriate antibiotics.

RosA and its derivative mutants (RosA^GRR1^ and RosA^GRR2^) were cloned in the integrative plasmid pHM2 under the control of a constitutive promoter (Pspac^c^), inserted between the *Eco*RI and *Hind*III restriction sites of pHM2. The *Pspac* promoter, normally inducible with IPTG, was amplified with the oligo pair CC1283/CC1284 to delete the operator site to convert it to a constitutive promoter. The RosA mutants were made by overlapping PCR. RosA^GRR1^ was constructed with oligo pairs CC2763/CC2686 and CC2685/CC2764. The RosA^GRR2^ mutant was made with oligo pairs CC2763/CC2684 and CC2683/CC2764. Subsequently, PCR products for each mutant and chromosomal DNA for the RosA WT were used as template for a new round of PCR with oligo pair CC2763/CC2764. The PCR fragments were cloned into pHM2-*Pspac^c^* between the *Hind*III and *Bam*HI restriction sites.

### *In vivo* RNA interactome

#### AMT *in vivo* cross linking

Bacteria were grown to the required O.D and 10 O.D _600 nm_ units were harvested by centrifugation (4000 g, 5 min, 4°C). Bacteria were resuspended in 2 ml PBS containing either no AMT (to identify background and levels of spurious interactions) or 0.7 mM AMT. Bacteria were incubated for 10 min at 37°C before being transferred to a six-well plate. The bacteria were exposed to UV 365 nm at 0.120 Jcm^–2^ for 10 min before being added to 1 ml of ice cold killing buffer (20 mM Tris–HCl [pH 7.5], 5 mM MgCl_2_, 20 mM Na-azide). The bacteria were harvested by centrifugation (4000 g, 5 min, 4°C). The supernatant was discarded and the pellet flash frozen in liquid nitrogen. We determined the *in vivo* RNA interactome of *B. subtilis* grown in M9 minimal media supplemented with 0.3% glucose at three points in the growth curve (exponential phase O.D._600 nm_ 0.5, stationary phase O.D._600 nm_ 1.4 and just after lysis had started to occur, and in LB at mid-exponential phase (O.D._600 nm_ of 1.0). A *Δfur* mutant (38) was prepared in LB at mid-exponential phase to increase expression of the Fur regulated sRNA FsrA. Samples were prepared in duplicate.

#### RNA extraction and formation of chimeras between interacting RNAs

The RNA was extracted by resuspending the cell pellet in 800 μl LETS buffer (10 mM Tris–HCl [pH 8.0], 50 mM LiCl, 10 mM EDTA, 1% sodium dodecyl sulfate [SDS]) and bead beating in a FastPrep using 0.1 μm glass beads for three rounds of 40 s. The tubes were transferred to ice in between cycles. The tubes were briefly spun to remove the bubbles created during bead beating. Two rounds of phenol/chloroform isoamyl alcohol extraction and one round of chloroform isoamyl alcohol extraction were carried out, before the addition of 10% (v/v) Na-acetate and 1 ml isopropanyl, and precipitation of RNA overnight at –20°C. The RNA was pelleted by centrifugation at maximum speed at 4°C and the pellet was washed with 70% ethanol before being air dried and resuspended in water. The RNA was quantified using the Qubit kit (Fisher Life Science). 10 μg of RNA was treated with Turbo DNase (Fisher Scientific) to remove contaminating DNA. Ribosomal RNA was removed using Ribozero (Illumina) according to the manufacturer's instructions. To form the chimeric RNAs between RNAs crosslinked with AMT, the protocol described by Sharma *et al.* was followed as described in the supplementary data (30). The only modification was the use of CircDNAligase (Epicentre) instead of CircRNAligase as this has been discontinued.

#### RNAseq

Following reversal of crosslinking at UV 254 nm, RNA was purified and resuspended in 10 μl H_2_O and processed through the TruSeq stranded total RNA library kit (150 bp) (Illumina) according to the manufacturer's instructions. The resulting libraries were sequenced on a MiSeq (Illumina).

#### Analysis

STAR aligner was used to map reads (Version STAR_2.6.0c_08–11) (39). This mapping tool is designed to analyse splicing of introns and exons, which is similar to what is created through the formation of chimeric reads where two different RNA fragments have been joined together. By identification of reads that map to different features (protein coding sequences, sRNAs, UTRs, transcripts for ncRNAs such as rRNA and tRNA, or transcribed intergenic regions) it is possible to identify RNA interactions. STAR aligner was set to single end read mode to map read 1 and read 2 separately, the chimeric detection mode activated, as this has been reported to be more sensitive to chimeric junctions. The allowed mismatches in mapping was set to default for STARaligner. The output from STAR was merged in to one Sam file, before being annotated using featureCounts within the package subread-1.6.3 in R, with all further statistical analysis also carried out in R. In our initial analysis, we found many reads mapped to unannotated features in the genome. To overcome this problem, we created new features for the unannotated regions of the genome and these are termed UA-start—stop in the data files.

Chimeric reads map to two different genomic features, whereas non-chimeric reads should only map to one feature. Exceptions are non-chimeric reads that map to more than one genomic feature because of repetitive genomic regions or across junctions between two neighbouring or overlapping genomic features. An interaction count table was generated of reads that mapped to more than one legitimate feature and thus are considered as interacting pairs (e.g. gene A-gene B). Chimeric reads that mapped to within one feature were recorded as intramolecular interactions (e.g. gene A-gene A) and included in the estimation of the total expression level of each individual gene. The interaction counts of two replicate samples of the same condition were added together to form a combined interaction count matrix. The matrix allowed statistical analysis of each interacting pair using a hypergeometric test. This statistical test compared the number of interaction read counts for each specific interaction with the number of other interaction read counts formed by each member of that pair with other RNAs, as well as with the total number of reads from the sample. All interaction pairs with a *P*-value below 0.05 were extracted as significant interactions. The P-adjusted value was calculated using the Benjamini-Hochberg procedure to control for the false discovery rate which was set at 0.05 (40).

To gain further confidence in pairs that form the most likely interactions, interacting pairs were further assessed by *in silico* prediction with IntaRNA2.0, which predicts the stability and binding position between two interacting RNAs (41,42). The gene and any untranslated region that has been identified associated with the gene of interest (12) were included in the prediction to take into account the transcriptional start and stop sites. If no UTR had been identified for an mRNA, the 50 bp up and downstream of the start and stop site were employed.

The analysis pipeline can be found in the following GitHub repository, https://github.com/StephenLi55/Bacillus_RNA_crosslinking_analysis.

### Proteomics analysis

Strains were grown to O.D._600 nm_ 1.0 in LB. 20 O.D. units were harvested and washed 3 X with PBS to remove media components. Cells were resuspended in 200 μl urea buffer (8 M urea, 50 mM Tris and 75 mM NaCl). 200 μl of urea buffer washed 0.1 μm beads were added to the cells before being disrupted using three rounds of bead beating for 40 seconds using a FastPrep. Cells were placed on ice between the three rounds of bead beating. The disrupted cells were then sonicated in a water bath for 15 min. Cell extracts were centrifuged at 15 000 × g for 5 min and supernatants used for protein quantification (Qubit protein assay kit). Protein reduction and alkylation was conducted by mixing 150 μg of total protein with 10 mM TCEP and 40 mM CAA, at 600 rpm, for 20 min at room temperature. After, proteins were predigested with 1.5 μg of rLysC (Promega) for 3 h at room temperature and samples diluted with 50 mM ammonium bicarbonate, 2 M urea final concentration. Protein digestion was performed with 1.5 μg of Trypsin (Promega) overnight at room temperature. The reaction was stopped by adding 1% TFA and 10 μg of peptides were desalted using StageTip (43).

Reversed phase chromatography was used to separate 1 μg of tryptic peptides prior to mass spectrometric analysis. The cell proteomes were analysed with two columns, an Acclaim PepMap μ-precolumn cartridge 300 μm i.d. × 5 mm, 5 μm, 100 Å and an Acclaim PepMap RSLC 75 μm i.d. × 50 cm, 2 μm, 100 Å (Thermo Scientific). The columns were installed on an Ultimate 3000 RSLCnano system (Dionex) at 40ºC. Mobile phase buffer A was composed of 0.1% formic acid and mobile phase B was composed of acetonitrile containing 0.1% formic acid. Samples were loaded onto the μ-precolumn equilibrated in 2% aqueous acetonitrile containing 0.1% trifluoroacetic acid for 8 min at 10 μl min^–1^ after which peptides were eluted onto the analytical column at 250 nl min^–1^ by increasing the mobile phase B concentration from 8% B to 25% over 90 min, then to 35% B over 12 min, followed by a 3 min wash at 90% B and a 15 min re-equilibration at 4% B.

Eluting peptides were converted to gas-phase ions by means of electrospray ionization and analysed on a Thermo Orbitrap Fusion instrument (Thermo Scientific). Survey scans of peptide precursors from 375 to 1500 *m*/*z* were performed at 120 K resolution (at 200 *m*/*z*) with a 2 × 105 ion count target. The maximum injection time was set to 150 ms. Tandem MS was performed by isolation at 1.2 Th using the quadrupole, HCD fragmentation with normalized collision energy of 33, and rapid scan MS analysis in the ion trap. The MS2 ion count target was set to 3 × 103 and maximum injection time was 200 ms. Precursors with charge state 2–6 were selected and sampled for MS2. The dynamic exclusion duration was set to 60 s with a 10 ppm tolerance around the selected precursor and its isotopes. Monoisotopic precursor selection was turned on and instrument was run in top speed mode.

Thermo-Scientific raw files were analysed using MaxQuant software v1.6.0.16 (http://www.maxquant.org) (44) against the UniProtKB *B. subtilis* database (UP000001570, 4260 entries). Peptide sequences were assigned to MS/MS spectra using the following parameters: cysteine carbamidomethylation as a fixed modification and protein N-terminal acetylation and methionine oxidations as variable modifications. The FDR was set to 0.01 for both proteins and peptides with a minimum length of seven amino acids and was determined by searching a reversed database. Enzyme specificity was trypsin with a maximum of two missed cleavages. Peptide identification was performed with an initial precursor mass deviation of 7 ppm and a fragment mass deviation of 20 ppm. The MaxQuant feature ‘match between runs’ was enabled. Label-free protein quantification (LFQ) was done with a minimum ratio count of 2. Data processing was performed using the Perseus module of MaxQuant v1.6.0.16 (45). Proteins identified by the reverse, contaminant and only by site hits were discarded. Only protein groups identified with at least two assigned peptides were accepted and LFQ intensities were log_2_ transformed. Significantly regulated proteins were identified in two rounds of analysis. First, a Student’s *t*-test (FDR 0.05) and a minimum difference of S0 = 0.1 was applied on all biological replicates. Second, a fine statistical analysis was applied using the same parameters as before but removing the outliers identified by principal component analysis and Pearson correlation test. The significantly regulated proteins were selected from both analyses.

### Plate reader experiments

Experiments to monitor promoter activity were carried out in a 96-well format in a BioTek Synergy Plate reader, with measurements every 10 min and analysed as described previously (36,37). Briefly, cultures were monitored for both optical density (O.D._600 nm_) and GFP fluorescence (excitation 485/20 nm, emission 528/20 nm). Background fluorescence from the isogenic WT control strain not expressing GFP was subtracted. Arbitrary units represent changes in promoter activity and were calculated using the equation (GFP^*t*^ − GFP^*t*−1^)/O.D._600*t*_ (where *t* represents a given time point at which fluorescence was measured, and *t* − 1 the preceding time point at which fluorescence was measured). Curves were smoothed using a moving average of three data points.

### RNA isolation and northern blotting

RNA was isolated from mid-log phase *B. subtilis* cells growing in the indicated medium by the RNAsnap method described in Stead *et al.* (46). Northern blots were performed as described previously (47). The S345/RosA riboprobe was transcribed *in vitro* using T7 RNA polymerase (Promega) and labelled with [α-^32^P]-UTP using a PCR fragment amplified with oligo pair CC2440/CC2441 as template. Oligos CC089, CC964 and CC875 were 5′ end-labelled with T4 polynucleotide kinase (PNK) and [γ-^32^P]-ATP and used to probe the *sucC, ppnkB* and RoxS RNAs, respectively.

### Mapping of 5′ ends by primer extension assays following Xrn1 digestion

Xrn1 depletion of 5′ monophosphorylated RNA was performed as described in Sinturel *et al.* (48).

### Quantification of sRNAs

RosA and RoxS RNAs were transcribed *in vitro* from PCR fragments amplified with the oligo pairs CC2406/CC2407 and CC1832/CC1833, respectively. Known quantities (in fmol) of *in vitro* transcribed RosA and RoxS RNAs, and 5 *μ*g total RNA isolated from WT cells were loaded on a denaturing 6% acrylamide gel. Oligos CC2347 and CC875 were 5′ end-labelled with T4 polynucleotide kinase (PNK) and [ γ-^32^P]-ATP and used to probe for RosA and RoxS, respectively, on Northern blots.

### Electrophoretic mobility shift assays (EMSA)

For EMSA assays, RosA and FsrA sRNAs were transcribed with T7 RNA polymerase *in vitro* from PCR fragments amplified with the oligo pairs CC2406/CC2407 and CC2492/CC2493, respectively. WT and CCCC to AAAA mutant variants of RoxS in CRR1 (RoxS^CRR1/4A^), CRR3 (RoxS^CRR3/4A^), or both, were transcribed from PCR fragments using oligo pair CC1832/CC1833, from plasmids 639, 640 and 641 containing the RoxS CRR mutants as templates (14). The RoxS CCC to GGG mutation in CRR1 (RoxS^CRR1/3G^) was transcribed from a PCR fragment amplified with oligo pair CC2831/1833. The FsrA CCCC to AAAA mutation in CRR1 (FsrA^CRR1/4A^) or the CC to AA mutation in CRR2 (FsrA^CRR2/2A^) were transcribed from PCR fragments amplified with oligo pairs CC2765/2493 and CC2783/2493, respectively. The FsrA CCC to GGG mutation in CRR2 (FsrA^CRR2/3G^) was transcribed from a PCR fragment amplified with oligo pair CC2858/2493.

The PCR fragment used to transcribe the short version of RoxS was synthesised with oligo pair CC2518/CC1833. The RosA RNA mutated in GRR1 (RosA^GRR1/4U^) and GRR2 (RosA^GRR2/4U^) were transcribed from PCR fragments amplified with overlapping upstream and downstream fragments containing the mutation (GGGG to TTTT) using oligo pairs CC2406/2686 and CC2685/2423 for the mutation in GRR1, and CC2406/2684 and CC2683/2423 to introduce the mutation in GRR2. The overlapping fragments were then assembled in a new PCR reaction with CC2406/2423. The RosA RNA mutated in GRR1 (RosA^GRR1/3C^) and GRR2 (RosA^GRR2/3C^) containing the mutation (GGG to CCC) were transcribed from PCR fragments amplified with overlapping upstream and downstream fragments using oligo pairs CC2406/2857 and CC2856/2423 for the mutation in GRR1, and CC2406/2830 and CC2829/2423 to introduce the mutation in GRR2. The overlapping fragments were then assembled in a new PCR reaction with CC2406/2423.

Before addition to EMSA assays, each RNA was individually heated for 3 min and cooled to room temperature for 10 min. A 15 μl reaction was prepared by mixing 5 pmol of RosA RNA with increasing concentrations of RoxS or FsrA RNA (2.5, 5 and 10 pmol) in 1× RNA binding Buffer (10 mM Tris pH 8; 50 mM NaCl; 50 mM KCl, 10 mM MgCl_2_) and incubated at 37°C. After 10 min of incubation, 10 μl of glycerol (stock solution 80%) was added and RNAs were loaded on a 6% non-denaturing polyacrylamide gel (acrylamide:bisacrylamide ratio 37.5:1). Following migration, RNA was transferred to a Hybond N+ membrane and hybridized with the S345/RosA radiolabelled probe close to the RosA terminator (CC2423).

### Strain competition experiment

Strains marked with appropriate antibiotic resistance cassettes were combined at a 1:1 ratio, inoculated at a starting O.D._600 nm_ and grown for 24 h in LB. To confirm starting ratios at a 1:1 ratio colony counts were performed on the initial inoculum. At 24 h cultures were serially diluted and plated on LB plates containing the relevant antibiotics to enable counting of each strain. Ratios of strains were calculated and Welch's *t* test was used to determine significance. An average of three technical replicates each containing three biological replicates was carried out for each combination of strains.

## RESULTS

### *In vivo* RNA crosslinking identifies known and unknown sRNA–RNA interactions

To identify new sRNA–mRNA interactions in *B. subtilis* we applied the LIGR-seq protocol (30) to *B. subtilis* cells growing in M9 minimal media supplemented with 0.3% glucose (exponential and transition phase) or in LB (WT and Δ*fur* mutant at exponential phase). The Δ*fur* mutant was included to increase the expression levels of the sRNA FsrA, the transcription of which is repressed by Fur (15). Cells were irradiated at 365 nm with the chemical crosslinker AMT (4′-aminomethyltrioxsalen). Biological replicates were prepared for each sample. RNAs were extracted, ligated, and non-crosslinked RNA was digested with RNase R. Crosslinks were reversed with 254 nm irradiation and RNA samples were subjected to high-throughput sequencing to detect chimeras formed by ligation (Supplementary Figure S1).

We designed and analysed the resulting RNA-seq data for chimeras using a customized pipeline, with a particular interest in identifying new interactions involving sRNAs (Supplementary Figure S1A). This included using STAR aligner, which is designed for mapping RNA-seq data containing spliced RNAs produced in eukaryotes, in single-end read mode with chimeric detection activated (39). This also enabled us to map chimeric reads where the ligation of the two fragments occurred close to the read ends.

In each of the eight individual samples analysed, many potential RNA–RNA interactions were identified using the customized pipeline (see Methods). In total we identified 10 642 different statistically significant interactions that mapped to different genes, where both parts were separated by over 2000 bp. As seen in Supplementary Figure S1B these interactions fitted into three different categories: mRNA–RNA, sRNA–RNA and stable RNA–RNA. Since our main goal was to identify new sRNA interactions, we focussed on this category for the purpose of this study. 593 different sRNA interactions were identified which were further split into three categories, sRNA–mRNA (448), sRNA–stable RNA (133) and sRNA–sRNA (12) (Supplementary Figure S1B). Four *B. subtilis* sRNAs have been well characterised; FsrA (15,21,49), RoxS (14,17), S1022 (36) and SR1 (50). Over half of the sRNA chimeras identified involved S1022, FsrA or RoxS. Of these RoxS and FsrA had the greatest number of known or predicted interactions (with CopraRNA (41,51), IntaRNA (41,42) or Target RNA target prediction tools (52)) (Supplementary Figure S1B, Supplementary Table S3A (FsrA) and 3B (RoxS)). Numerous previously identified mRNA targets of FsrA (*gltD*, *resABC*, *qcrB*, *sdhC*, *citB, gltAB, leuABCD* and *lutABC*) were identified in at least one of our growth conditions (Supplementary Table S3) (15,21). Although only a few known targets of RoxS (*citZ* and *etfB)* (14) and none of the three well-characterized targets of RoxS in *B. subtilis* (*ppnKB*, *sucCD* and *yflS*) were found in the data-set, this could be explained by the fact that growth conditions (lacking malate) were probably not optimal for RoxS expression (17). In total, we identified 108 and 89 interactions for FsrA and RoxS, respectively, including mRNAs, sRNAs and stable RNAs. Of these interactions, 47 for FsrA and 44 for RoxS also had P-adjusted values below 0.05. The IntaRNA prediction for each interaction is also shown (Supplementary Table S3).

**Figure 1.**
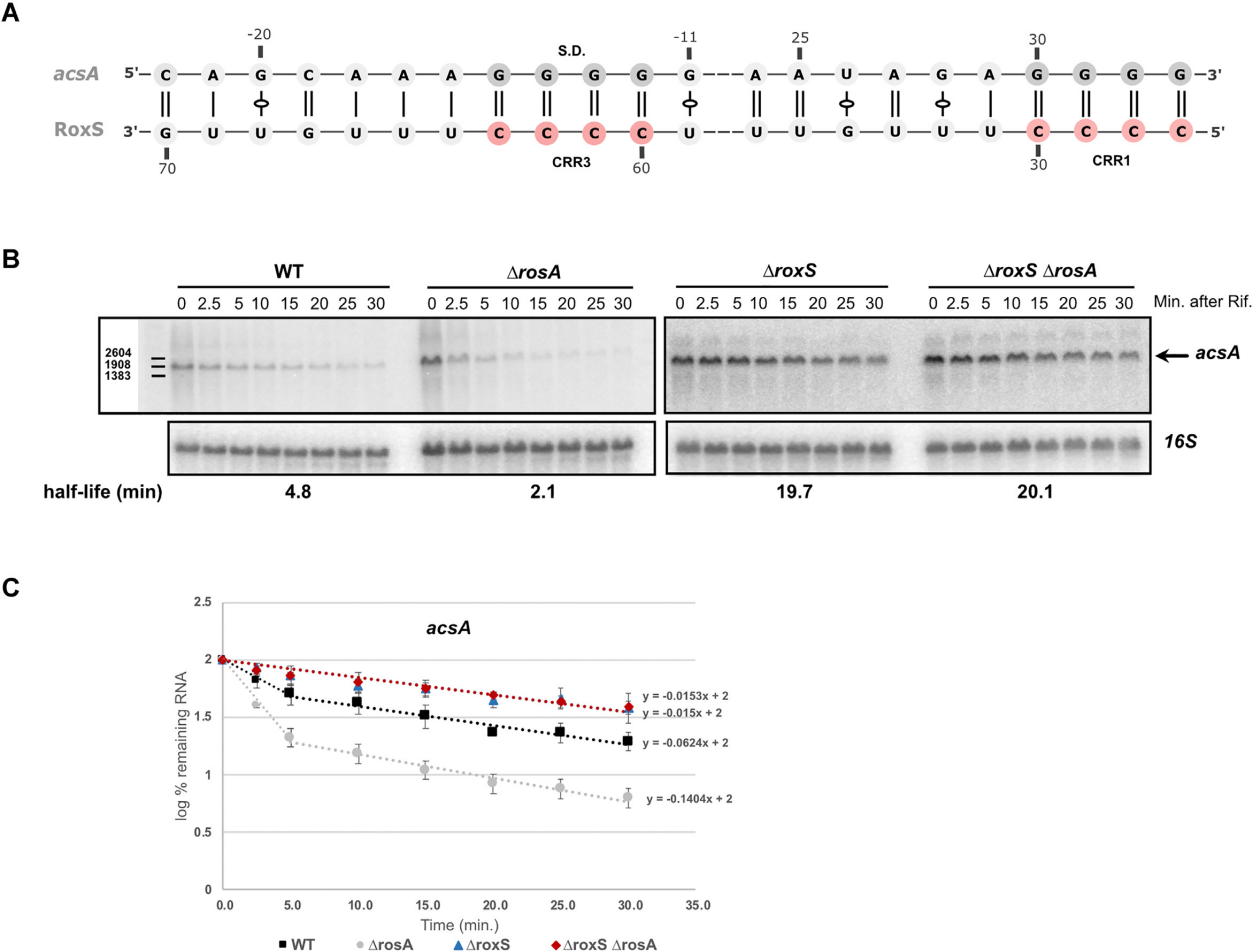
Deletion of RosA and/or RoxS alters the turnover rate of *acsA* mRNA. (**A**) Proposed base-pairing between RoxS and the *acsA* mRNA. (**B**) Northern blot of total RNA isolated from WT, *ΔrosA*, *ΔroxS* and *ΔrosA ΔroxS* strains probed for the *acsA* mRNA at times after addition of rifampicin (min. after Rif). The blot was re-probed for 16S rRNA as a loading control. Calculated half-lives are shown beneath the autoradiographs and are the average of at least two experiments. (**C**) Graph of RNA decay curves showing the log percent RNA remaining (average of two experiments), with standard deviation for each time point after rifampicin addition used to calculate half-lives reported in panel B. For biphasic curves, half-lives were calculated from the initial slope of the curve.

Several potential new targets with a probable link to iron metabolism were identified for FsrA. For example, we identified many chimeras between FsrA and the *yydF* mRNA, encoding a secreted peptide that controls LiaRS activity (53). The gene downstream of *yydF* in this operon, *yydG*, encodes a protein that contains an iron-sulphur (Fe-S) cluster and is part of a protein complex required to process YydF into a functional peptide. In the case of RoxS, which is known to regulate the expression of several RNAs encoding proteins involved in central metabolism (e.g. enzymes of the TCA cycle), such as citrate synthase (CitZ) (14), we detected a new interaction between RoxS and the *acsA* mRNA encoding acetyl-CoA synthetase that converts acetate to acetyl-CoA, which can be incorporated in the TCA cycle. Potential base-pairing with the SD region of this gene was predicted by IntaRNA (Figure 1A), suggesting that regulation could occur though inhibition of translation, with subsequent indirect effects on mRNA stability. To confirm an effect of RoxS on *acsA* mRNA half-life, we performed a northern blot experiment after rifampicin treatment to block new transcription (Figure 1B and C). In agreement with our hypothesis, the stability of the *acsA* mRNA increased ∼4-fold in the *ΔroxS* mutant strain compared to the WT (4.8 min half-life in WT versus 19.7 min in the *ΔroxS* strain).

The above data confirm the ability of the LIGR-seq technique to identify new potential sRNA–mRNA interactions in bacteria. This method is complementary to those focusing on individual RNAs such as MAPS (54) or those based on specific RNA binding proteins such as RIL-seq (23) or CLASH (25), but has the advantage of not requiring prior knowledge about the different partners.

### Identification of a novel robust sRNA–sRNA interaction

Analysis of the RNA interactome allowed us to map 12 potential sRNA–sRNA interactions. Four of these interactions involved FsrA, RoxS or S1022 (Supplementary Figure S1B). The most represented chimera pairs were those of FsrA and RoxS with the predicted sRNA S345 and S346 (annotated as the 3′ UTR of S345) (see below) (Supplementary Figure S1B). The interaction was not only of strong statistical significance, but was also found in multiple growth conditions (Supplementary Table S3). As robustly described sRNA–sRNA interactions are rare (for other examples see (55–57)) we sought to characterize this pair further.

### RosA is a highly processed sRNA

**Figure 2.**
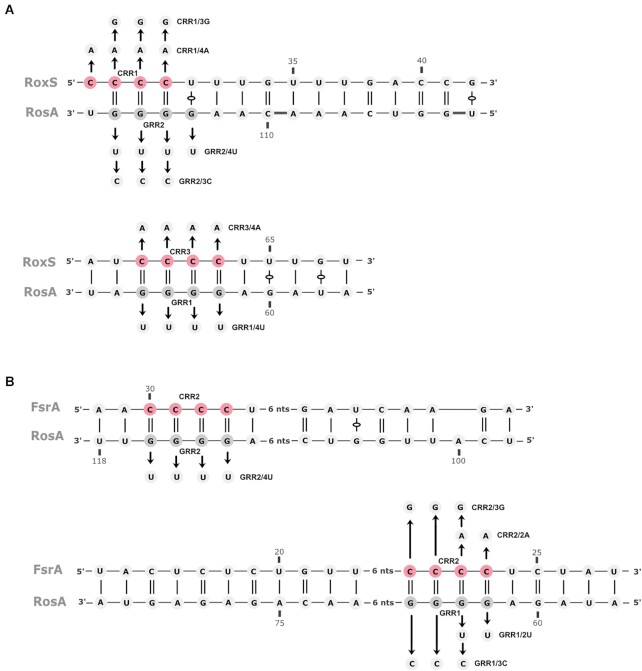
Predicted interactions between RosA and RoxS or FsrA. The interaction between RosA and RoxS (**A**) and FsrA (**B**) was predicted by the IntaRNA web server. The C-rich regions of RoxS and FsrA are coloured in red. The G-rich regions of RosA are coloured in dark grey. Arrows indicate mutagenized residues to construct RosA mutants in GRR1 (RosA^GRR1/4U^, RosA^GRR1/2U^ and RosA^GRR1/3C^) and GRR2 (RosA^GRR2/4U^ and RosA^GRR2/3C^), RoxS mutants in CRR1 (RoxS^CRR1/4A^ and RoxS^CRR1/3G^) and CRR3 (RoxS^CRR3/4A^), and FsrA mutants in CRR2 (FsrA^CRR2/2A^ and FsrA^CRR2/3G^), used in Figure 5.

**Figure 3.**
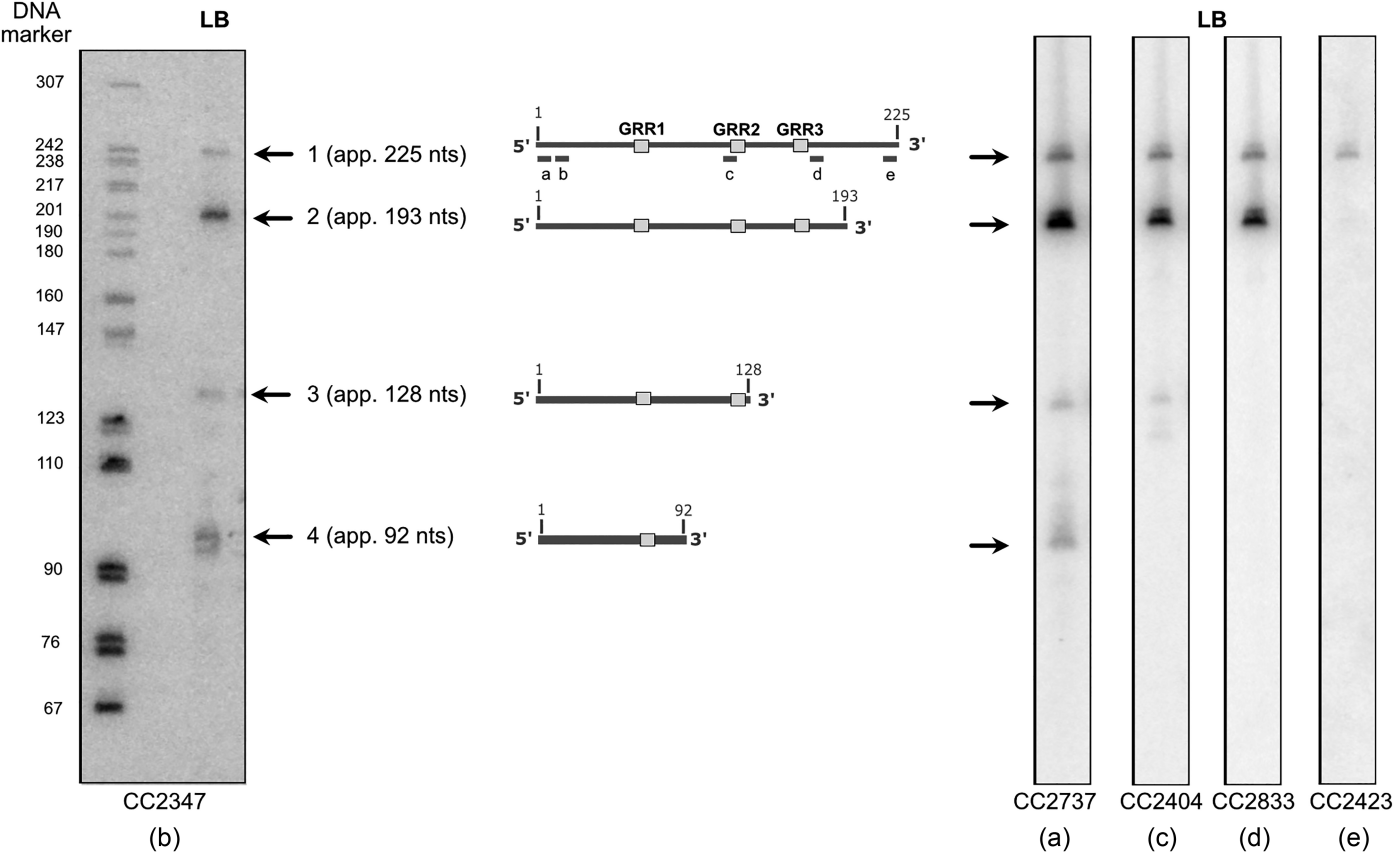
RosA is a highly processed sRNA. Northern blot of total RNA isolated from WT grown in LB and probed for the RosA sRNA with five different oligo probes: left, oligo CC2347 (b) (beginning 30 nts from the 5′-end); right, oligo CC2737 (a) complementary to the extreme 5′ end of RosA, CC2404 (c), CC2833 (d) and CC2423 (e) complementary to the terminator of transcription showing that the four RosA species have the same 5′-end. The pBR322 plasmid digested with *Msp*I and labelled with ATP-γ-P^32^ was used as a DNA ladder to estimate the sizes of the four RosA species. A standard curve of log size vs distance migrated was generated using the DNA marker and used to calculate RNA sizes, taking into account the supplementary oxygen on RNA (left lane). Species 1 to 4 are represented in the middle of the figure. The G-rich regions (GRR1, 2 and 3) are indicated by grey boxes.

In a previous study, the S345 segment was predicted to have a sigma A-dependent promoter, with a Rho independent terminator located in S346, producing an RNA of approximately 229 nts (12). The predicted sequence of S345/S346, which we renamed RosA (see below), has three G rich regions (GRRs) (Supplementary Figure S2) with potential complementarity to the C-rich regions (CRRs) of FsrA and RoxS that have been shown to be involved in the interactions with their mRNA targets (Figure 2). The GRR1 and GRR2 sequences are predicted to be in single stranded regions directly accessible for interactions with C-rich sRNAs. We confirmed this structure *in vitro* by DMS probing (Supplementary Figure S2). As a first step in characterising RosA, we performed Northern blots on total RNA isolated from cells grown in LB to mid-exponential phase, probed with an oligo complementary to a region starting 30 nts from the 5′ end of RosA. In these conditions, we detected four main products, showing that this RNA is highly processed (Figure 3, left, probe b). The size of the largest product was ∼225 nts, in agreement with the size of S345 + S346 combined (12). The 5′-end of RosA suggested by the LIGR-seq data was confirmed by primer extension (Supplementary Figure S3). The predicted sigma-A promoter proposed by Nicolas *et al.* (12) fits perfectly with this mapped 5′ end.

The three smaller species were approximately 193 nts, 128 nts and 92 nts for bands 2, 3 and 4, respectively. A Northern blot performed with an oligoprobe complementary to the extreme 5′ end of RosA (probe a) gave a similar pattern (Figure 3, right). We deduced that these forms of RosA have the same 5′ end, and that species 2, 3 and 4 are processed from the primary transcript at 3′ proximal sites. Our LIGR-seq data showed numerous 3′ truncations of RosA. We were unable to map primer extension arrests corresponding to the cleavage events yielding species 2, 3 and 4, presumably due to the instability of the resulting 3′ fragments. The approximate positions for the cleavage sites generating species 2, 3 and 4 are indicated in Supplementary Figure S2 and based on the size determined by Northern blot. Reprobing of the Northern blot with several probes (c, d and e) close to the deduced 3′ ends of the different species confirmed these results (Figure 3, right).

We attempted to identify the RNases involved in RosA processing by testing the effect of several endo- (RNase Y, RNase III) and exoribonuclease mutations (RNase J1, PNPase, RNase R, RNase PH, YhaM) on RosA processing *in vivo* (Supplementary Figure S4A). Surprisingly, none of the ribonucleases tested were individually responsible for the generation of species 2, 3 or 4 of RosA. However, species 1 and 3 increased in the absence of the endoribonuclease Y to the detriment of species 2 and 4, suggesting that RNase Y is at least partially responsible for the cleavages that yield the latter two species. The level of species 2 was sensitive to the 3′-5′ exoribonuclease PNPase, suggesting that its degradation depends on this exoribonuclease. Species 4 disappeared in strains lacking PNPase or RNase PH (a 3′-5′ exoribonuclease related to PNPase). In these two mutant strains (*Δrph*, *Δpnp*), a slower migrating band appeared, suggesting that species 4 is produced by the trimming of few nucleotides of a larger species by RNase PH and PNPase at its 3′-end. Furthermore, in the strain deleted for the four 3′-5′ exoribonucleases (PNPase, RNaseR, YhaM and RNase PH), a new species also appeared just above species 3, showing that yet other unstable intermediates are trimmed by 3′-5′ exoribonucleases to produce species 3 (or 4). These results show that the different 3′ ends of RosA are generated by a highly complex pathway involving multiple endo- and exoribonucleases, some of which remain to be identified.

Species 2 and 3 increased visibly in the absence of the 5′-3′ exoribonuclease RNase J1, suggesting that these two RNAs can be degraded directly from their 5′ ends. This was confirmed by rifampicin experiments showing a stabilization of species 2 and 3 (5-fold and almost 3-fold respectively) in a *ΔrnjA* strain compared to the WT strain (Supplementary Figure S4B and C).

### RosA is subject to carbon catabolite repression

In comparing the stabilities of the various RosA species in conditions similar to those used in the crosslinking experiment (LB and in M9 minimal medium + glucose), we noticed that while the level of the RosA RNA was higher at T0 in LB than in M9 at mid-exponential phase, the half-lives of species 1 and 2 of RosA were similar (Supplementary Figure S4B). This suggested that the lower levels of RosA in M9 + glucose were most likely due to transcriptional regulation. The DBTBS server (58) predicted a binding site for the transcriptional regulator CcpA between -1 to + 12 relative to the mapped 5′ end of RosA (Supplementary Figure S3). CcpA mediates carbon catabolite repression in *B. subtilis*, repressing catabolic genes and activating genes involved in the excretion of excess carbon (59). The prediction of a CcpA binding site in the promoter region of RosA was corroborated by Marciniak *et al.* (60). Furthermore, the expression profile of RosA (S345) in the 104-condition tiling array data for *B. subtilis* was very similar to known members of the CcpA regulon, such as MalA, AcoA and AbnA, consistent with the idea that RosA is a CcpA-regulated sRNA (12).

**Figure 4.**
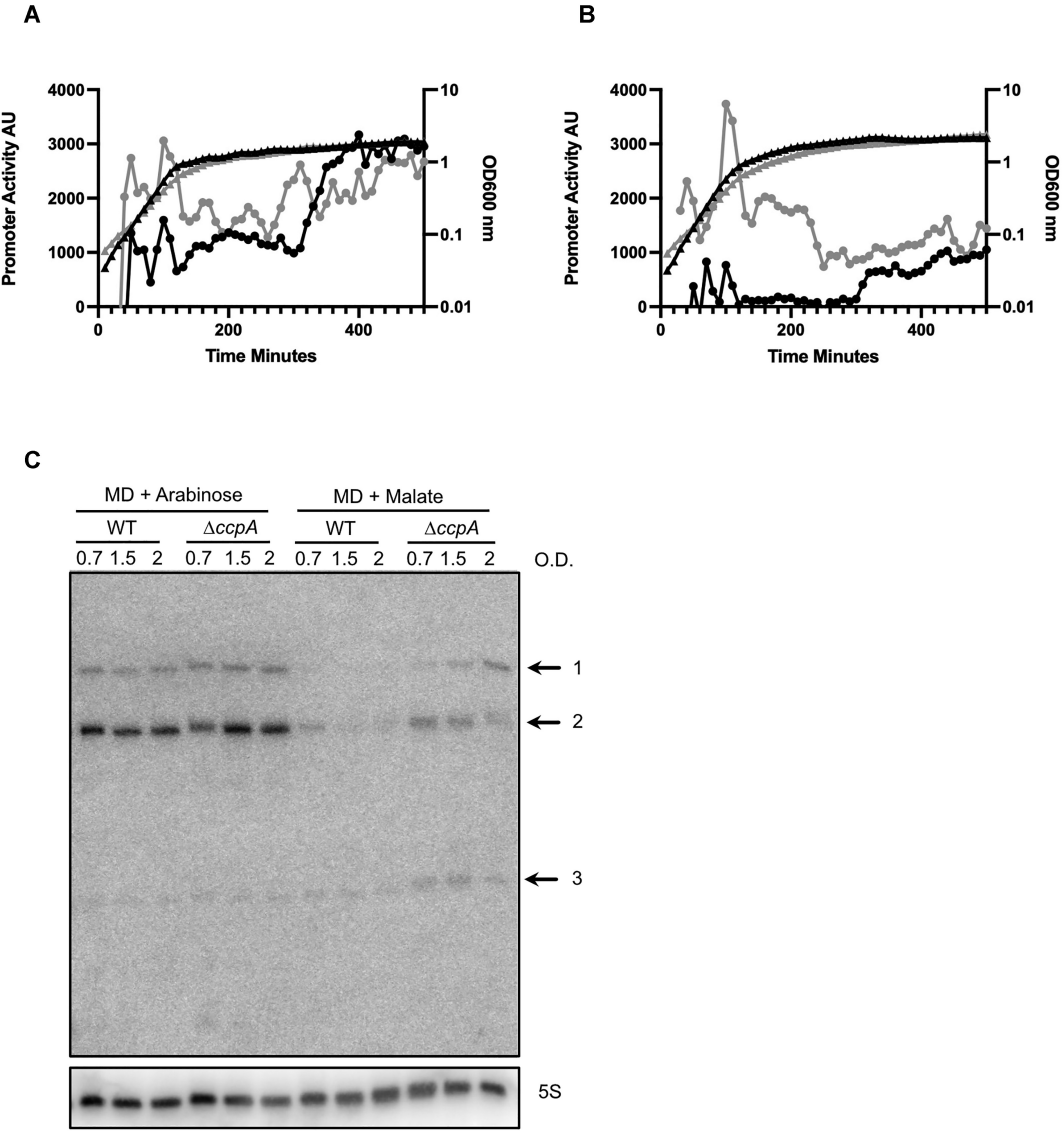
RosA is a CcpA-regulated sRNA. (**A**) Assay of the *RosA* promoter fused to GFP in WT and Δ*ccpA B. subtilis* strain grown in LB and (**B**) LB + 0.3% glucose. Promoter activity is presented in arbitrary units (AU). The black line with triangles corresponds to the WT growth curve and the grey line with triangles to the Δ*ccpA* growth curve. The black line with circles corresponds to the promoter activity of RosA in the WT, and the grey line with circles to the promoter activity in Δ*ccpA*. Experiments were done in triplicate. (**C**) Northern blot of RosA in WT and *ΔccpA* mutant strain grown in MD medium with arabinose (1%) or malate (1%) as carbon sources. RNA was extracted at different optical densities (O.D.) during growth, as indicated.

To confirm that CcpA indeed regulates RosA *in vivo*, we fused the promoter of RosA to GFP using the BaSysBioII vector (37). We monitored expression of this fusion in WT *B. subtilis* and in an isogenic mutant lacking the *ccpA* gene. No difference in P*rosA*-GFP expression could be seen between the WT and the Δ*ccpA* strain in LB medium (Figure 4A). Addition of 0.3% (w/v) glucose to the medium resulted in repression of *rosA* promoter activity in the WT strain, whereas in the absence of *ccpA* the *rosA* promoter remained active, as predicted (Figure 4B). We also performed a Northern blot experiment where we directly measured the levels of RosA sRNA in a defined medium containing malate or arabinose (1%) (Figure 4C). RosA sRNA levels were similar in WT and *ΔccpA* mutant strains grown in arabinose where CcpA is inactive. However, in the presence of the catabolite repressing carbon source malate, RosA sRNA levels were repressed in the WT strain and this repression was alleviated in the *ΔccpA* mutant strain. These results clearly show that RosA is transcriptionally regulated by CcpA.

### RoxS interacts directly with RosA *in vitro*

The base-pairing prediction between RoxS and RosA by IntaRNA (42) incorporates both GRR1 and GRR2 of RosA, and CRR3 and CRR1 of RoxS, respectively (Figure 2A). In contrast, for FsrA, two alternative interactions with RosA were predicted. Both predictions involved the CRR2 region of FsrA with either the GRR1 or the GRR2 sequence of RosA (Figure 2B). All predictions include long stretches of interacting nucleotides, suggesting these two RNA pairs can form stable duplexes.

**Figure 5.**
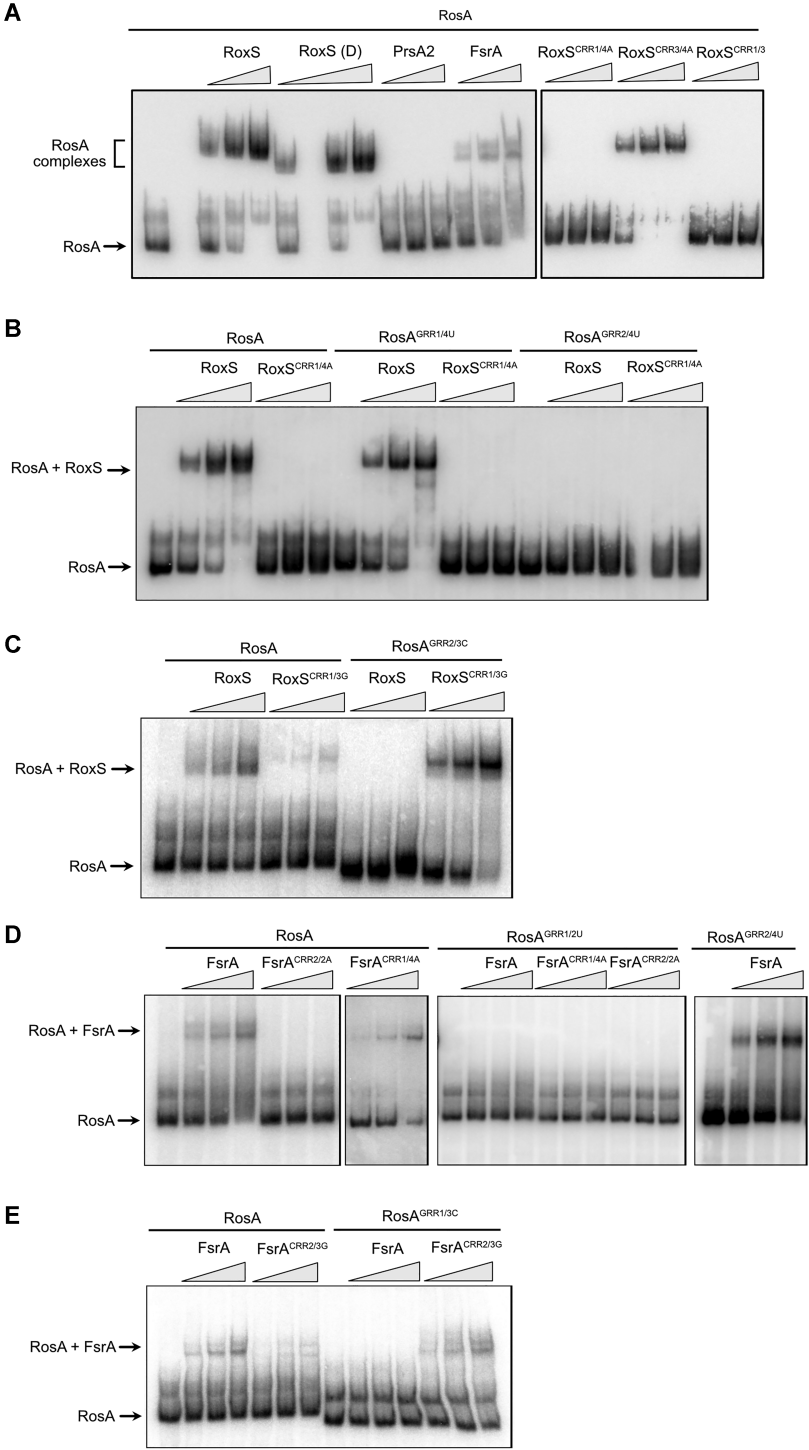
RosA interacts directly with RoxS and FsrA sRNAs. (**A**) Left: Electrophoretic mobility shift assays (EMSA) of RosA (full length; FL) with RoxS (FL), the cleaved form of RoxS (D), FsrA and PrsA2 RNA (negative control from *L. monocytogenes*). Right: EMSA of RosA with RoxS mutated in CRR1 and/or CRR3 (where the four C’s were replaced by four A’s; see Figure 2B). The mutant constructs are referred to as RoxS^CRR1/4A^ and RoxS^CRR3/4A^, respectively. Lanes 2 and 7 are empty. (**B**) EMSAs of WT RosA (FL) or RosA mutants in GRR1 or GRR2 (where the four G’s were replaced by four U’s) with WT RoxS or the RoxS^CRR1/4A^ complementary mutant. The RosA mutant constructs are referred to as RosA^GRR1/4U^ and RosA^GRR2/4U^, respectively. (**C**) EMSAs of WT RosA (FL) or the RosA GRR2 mutant, where 3 G’s were replaced by three C’s (RosA^GRR2/3C^), with WT RoxS or the RoxS^CRR1/3G^ complementary mutant. (**D**) EMSAs of WT RosA(FL), RosA^GRR1/2U^ (where two G’s were replaced by two U’s) or RosA^GRR2/4U^ (see Figure 2B) with WT FsrA, the FsrA^CRR1/4A^ mutant, or the FsrA^CRR2/2A^ mutant (complementary mutation to RosA^GRR1/2U^). In each experiment, 5 pmol of RosA RNA was incubated with an increasing concentration (2.5, 5 and 10 pmol) of RoxS, PrsA2 or FsrA RNA, as indicated. (**E**) EMSAs of WT RosA (FL) or RosA GRR1 mutants where 3 G’s were replaced by three C’s (RosA^GRR1/3C^) with WT FsrA or the FsrA^CRR2/3G^ complementary mutant.

We performed electrophoretic mobility shift assays (EMSA) to confirm the potential interactions between RosA and RoxS or FsrA (Figure 5). RosA was first mixed with increasing concentrations of RoxS, the cleaved form of RoxS (RoxS (D)) previously identified to regulate the *sucCD* operon (14), or FsrA and loaded on a non-denaturing acrylamide gel. The results show that RoxS and RoxS (D) bound very efficiently to RosA, producing a sharp band of higher molecular weight (Figure 5A and B). Complex formation between FsrA and RosA was also observed (Figure 5A, D and E). Two bands of higher molecular weight were visible, with their close proximity probably being more consistent with two alternative structures of the duplex than binding of two FsrA molecules to RosA. The 5′ UTR of PrsA2 mRNA of *Listeria monocytogenes* was used as a negative control and no shift was found with RosA as expected (Figure 5A). These results confirm that RoxS and FsrA sRNAs are both able to bind the RosA sRNA.

To test the base-pairing prediction between RoxS CRR1 and RosA GRR2 on the one hand, and RoxS CRR3 and RosA GRR1 on the other, we made mutations in these different regions (Figure 2). A mutation of RoxS CRR1 in which the four C residues were replaced by four A’s (RoxS^CRR1/4A^), completely abolished the binding of RoxS to RosA (Figure 5A). In contrast, the same mutation of CRR3 (RoxS^CRR3/4A^) had no effect on RosA binding. A double mutant of CRR1 and 3 (RoxS^CRR1/3)^ behaved like the single RoxS^CRR1/4A^ mutant. This result suggests that the predicted base-pairing between GRR2 and CRR1 is the key interaction for binding of RosA to RoxS. To confirm this, we first replaced the four G’s of RosA GRR1 or GRR2 by four U’s (RosA^GRR1/4U^ or RosA^GRR2/4U^, respectively) and tested the ability of these mutant RNAs to bind WT RoxS or RoxS carrying the complementary CRR1/4A mutation (Figure 5B). As anticipated, WT RoxS failed to bind the RosA^GRR2/4U^ mutant, but bound the RosA^GRR1/4U^ variant efficiently. Curiously, RoxS bearing the compensatory mutation in CRR1 (RoxS^CRR1/4A^) failed to interact with the complementary RosA^GRR2/4U^ mutant. The same was true for reciprocal mutations in RosA and RoxS (i.e. CRR1 mutated to four U’s (RoxS^CRR1/4U^) and GRR2 mutated to four A’s (RosA^GRR2/4A^) (Supplementary Figure S5). We surmised that the replacement of the four G–C base-pairs by four A–U pairs probably reduced the strength of this interaction sufficiently to prevent base-pairing between the two RNAs. To test this hypothesis, we constructed two additional mutants in which G–C base-pairs were switched to C–G, i.e. a mutation in the RosA RNA where 3 G’s of GRR2 were replaced by 3 C’s (RosA^GRR2/3C^) and the compensatory mutation in RoxS where 3 C’s of CRR3 were replaced by 3 G’S (RoxS^CRR3/3G^) (Figure 2A). As expected, WT RoxS was unable to interact with the RosA^GRR2/3C^ variant but RoxS^CRR3/3G^ bearing the compensatory mutation could (Figure 5C). This experiment confirms the critical importance of G-C base-pairs in the interaction between RoxS and RosA, and that they cannot be replaced by weaker A–U pairs, even though the total number (15) of potential base-pairs remains considerable (Figure 2A).

We also confirmed the interaction between the GRR2 sequence of RosA and the CRR1 region of RoxS by DMS probing. Indeed, in presence of RoxS, the DMS reactivity decreased for A residues (A107–A112) located just upstream of GRR2 and predicted to base-pair with RoxS (Supplementary Figure S2, centre panel). Moreover, we observed a reverse transcriptase stop at the last G of GRR2 due to the strong base-pairing of RoxS in this region. A similar arrest of the reverse transcriptase in the presence of RoxS was observed previously for *sucCD* and *ppnkB* mRNA (14).

To confirm the key regions of interaction between FsrA and RosA, we also performed EMSA assays with RosA and FsrA mutants. The 4 C’s of the CRR1 sequence of FsrA were first mutated to four A’s (FsrA^CRR1/4A^) and 2 C’s of CRR2 to 2 A’s (FsrA^CRR2/2A^) (Figure 2B). Mutation of FsrA CRR1 did not affect FsrA binding to RosA, while the FsrA^CRR2/2A^ mutation abolished the interaction (Figure 5D), confirming that FsrA CRR2 is more important for pairing. This was confirmed by mutation of the complementary GRR1 and GRR2 regions of RosA (RosA^GRR1/2U^ and RosA^GRR2/4U^). WT FsrA still bound to RosA^GRR2/4U^ but failed to form a complex with RosA^GRR1/2U^. Together, these results show that the CRR2 sequence of FsrA and the GRR1 region of RosA, the site of the most stable interaction predicted by the IntaRNA program (42), are most likely to be involved in the pairing between the two RNAs. However, as observed with RoxS, we were unable to restore the interaction between FsrA^CRR2/A^ and the complementary mutant RosA^GRR1/2U,^ again suggesting that the four GC base-pairs are crucial to allow the interaction between the two RNAs. We also mutated RosA GRR1, replacing three G’s with 3 C’s (RosA^GRR1/3C^) and FsrA CRR2, where the three C residues were mutated to three G’s (FsrA^CRR2/3G^). As observed for RoxS, FsrA^CRR2/3G^ only interacted with RosA^GRR1/3C^ bearing the compensatory mutation that maintains the number of G–C pairs (Figure 5E).

### RosA destabilises RoxS and promotes its processing to its truncated form

**Figure 6.**
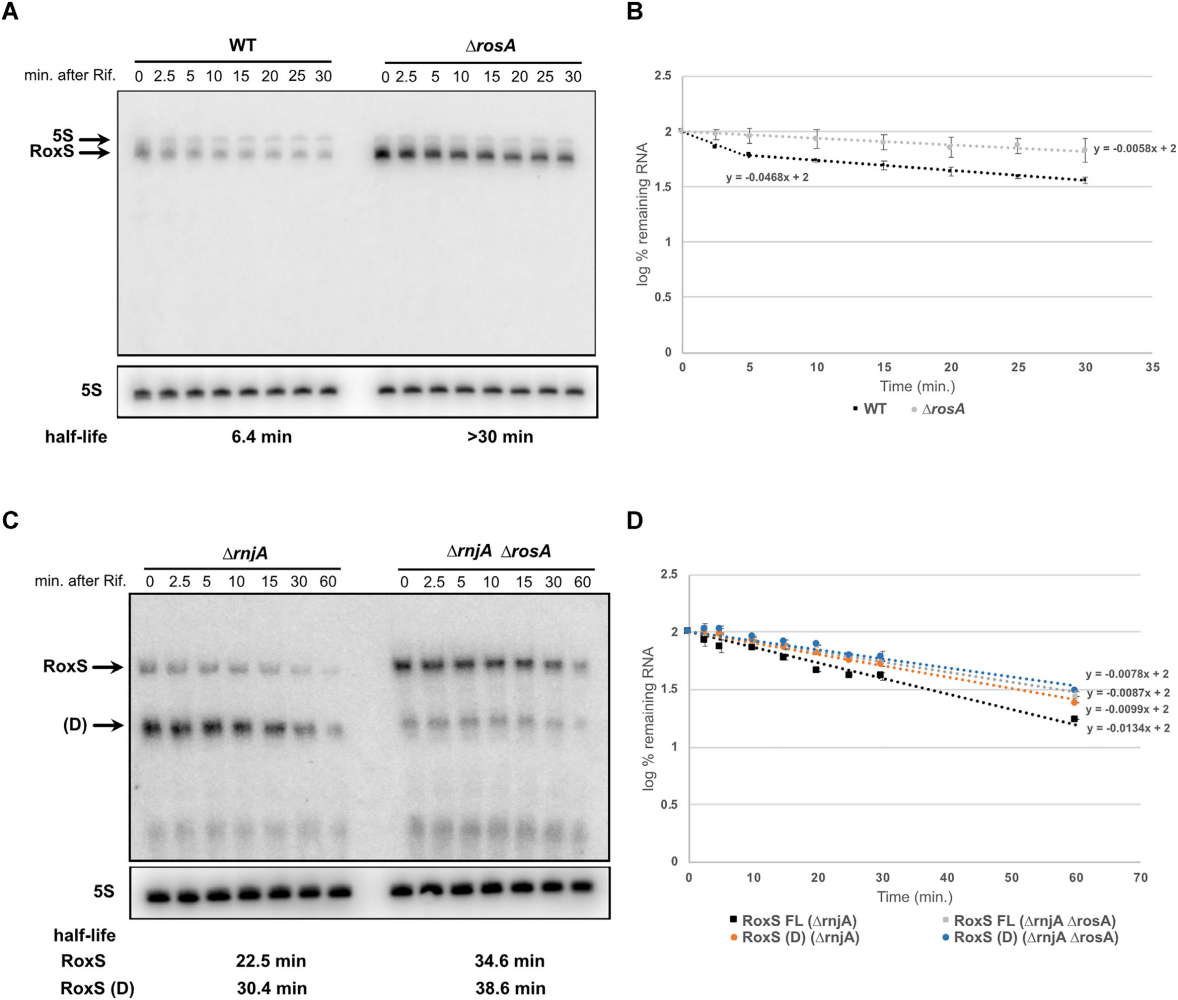
Deletion of RosA alters the turnover rate of RoxS. (**A**) Northern blot of total RNA isolated from WT and *ΔrosA* strains probed for the RoxS sRNA at times after addition of rifampicin (Rif). The blot was re-probed for the 5S rRNA as a loading control. (**B**) Graph of RoxS RNA decay curves in WT and *ΔrosA* strains showing the log percent RNA remaining with standard deviation calculated from two independent experiments (biological replicates) for each time point after rifampicin addition. For biphasic curves, half-lives were calculated from the initial slope of the curve. (**C**) Northern blot of total RNA isolated from *ΔrnjA* and *ΔrnjA ΔrosA* strains probed for the RoxS sRNA at times after addition of rifampicin (Rif). The blot was re-probed for 5S rRNA as loading control. RoxS (FL): Full length transcript, RoxS(D): truncated form of RoxS. Half-lives are given under each autoradiogram. (**D**) Graph of RoxS RNA decay curves in WT and *ΔrosA* strains showing the log percent RNA remaining with their standard deviation calculated from two independent experiments (biological replicates) for each time point after rifampicin addition. The slope of the graph was determined using linear regression function in Excel. Half-life (*t*_1/2_) was calculated from the equation: *t*_1/2_ = –log_10_(2)/slope.

To determine whether RosA had an effect on RoxS levels or stability *in vivo*, we measured the rate of RoxS RNA degradation before and after the addition of rifampicin to WT and *ΔrosA* mutant strains. The experiment was carried out in LB, since RosA is expressed at higher levels in this medium. Samples were taken over a time course of 0 to 30 min and the RNA analysed by northern blot. RoxS expression was significantly higher in the absence of RosA (Figure 6A, B). In the WT strain, the half-life of RoxS is bi-phasic as previously observed (14) suggesting that two populations of RoxS co-exist. The half-life of the rapidly decaying population is around 6 min. Interestingly, in the absence of RosA, the decay of RoxS is monophasic and its half-life is greater than 30 min. This result shows that the rapidly decaying population of RoxS is probably a consequence of its base-pairing with RosA, which stimulates its degradation. The increase in RoxS half-life (5-fold) presumably also explains its higher steady state levels in the *ΔrosA* mutant at the zero time point.

We previously showed that RoxS is processed by RNase Y to remove the first 20 nts of the transcript, producing a shorter more unstable version of the sRNA called RoxS (D) that is better able to regulate some targets than the full-length form (14). We therefore asked whether RosA had an effect on RNase Y processing of RoxS. RoxS (D) can be readily detected in a strain lacking the 5′-3′ exoribonuclease RNase J1 (encoded by *rnjA*), since this RNase is involved in the rapid degradation of the processed species. We therefore performed Northern blots on cells treated with rifampicin to compare the relative amounts and half-lives of RoxS and RoxS (D) in *ΔrnjA* versus *ΔrnjA ΔrosA* cells. As observed previously RoxS (D) was the predominant form of RoxS present in the *ΔrnjA* strain. In contrast, in the double *ΔrnjA ΔrosA* mutant, full length RoxS was the predominant form (Figure 6C, D). An alternative pathway of the degradation of RoxS via the direct attack of the full-length RNA by RNase J1 has also been characterized (14). This was confirmed here, where the full-length RoxS was stabilized in the *ΔrnjA* strain compared to the WT strain (22.5 min *vs* 6.4 min half-life) (Figure 6A, C). Interestingly, deletion of RosA in the *ΔrnjA* background only modestly increased the half-life of full length RoxS (22.5 min in *ΔrnjA* compared to 34.6 min in *ΔrnjA ΔrosA* mutant), in contrast to the 3.5-fold effect of the RosA deletion in an *rnjA^+^* background (Figure 6A, C). Thus, on the one hand, RosA increases the efficiency of processing of RoxS to its truncated form by RNase Y and, on the other, promotes the degradation of the full-length RoxS by RNase J1. In contrast, the deletion of RosA has little impact on the stability of FsrA in LB, with only a 50% difference in half-life observed in the *ΔrosA* strain (Supplementary Figure S6).

To confirm that the effect of RosA on RoxS mRNA stability was linked to their interaction *in vivo*, we introduced WT and mutated versions of RosA (RosA^GRR1/4U^ or RosA^GRR2/4U^) into the *amyE* locus in a strain deleted for RosA at its native site and examined their effects on RoxS levels by Northern blot. The transcription of RosA was controlled by a constitutive variant of the *Pspac* promoter (*Pspac^c^*) and cells were grown in rich medium (2xTY) with malate to maximize RoxS expression (17). As expected, when RosA was expressed constitutively (pHM2-RosA), RoxS was destabilized >5-fold compared to the control strain with the empty vector (pHM2) (Supplementary Figure S7). Similarly, the RosA GRR1 mutant (RosA^GRR1/4U^) also dramatically reduced RoxS levels. The RosA^GRR2/4U^ mutant was unable to impact RoxS levels, confirming the importance of the GRR2 sequence for RoxS destabilisation. Moreover, band 3 of RosA, corresponding to a species stabilized by RoxS (see below) was absent in strains lacking RosA or expressing the RosA^GRR2/4U^ mutant, consistent with a lack of interaction between RoxS and the RosA^GRR2/4U^ RNA.

Overall, our *in vivo* and *in vitro* data support the hypothesis that the primary interaction between RosA and RoxS occurs between CRR1 of RoxS and GRR2 of RosA, generating a longer and stronger hybrid than the potential interaction between RoxS CRR3 and RosA GRR1 (Figure 2A). However, we cannot exclude the possibility that the CRR3/GRR1 interaction occurs subsequently.

### RoxS plays a role in the processing of RosA

In the previous section, we showed that RosA is important for the processing of RoxS to RoxS (D). We therefore wondered whether the converse was also true, *i.e.*, whether RoxS had an effect on the processing of RosA. We analysed the expression pattern and degradation rates of the different RosA species in the presence and absence of RoxS. The RosA pattern was very similar in the presence or absence of RoxS, except for RosA species 3, which was completely absent (Supplementary Figure S8). As suggested above, the size of species 3 (∼125 nts) is consistent with an RNA that extends from the mapped 5′ to the end of the predicted duplex with RoxS with GRR2 around nt 116. The duplex could potentially protect the 3′ end of species 3 from 3′ exoribonucleases, consistent with its relatively long half-life (9 min) compared to species 1 and 2 (∼1.5 min). In the absence of the protective effect of RoxS, species 3 would be degraded very rapidly.

To better understand the physiological relevance of the RoxS/RosA interaction *in vivo*, we calculated the relative amount of RosA and RoxS present in the cells grown in LB. In 5 μg of total RNA, RosA and RoxS were present at approximately equimolar amounts (10 fmol each; Supplementary Figure S9). This result shows that there is sufficient RosA in the cell to completely titrate all RoxS present in the cell under equilibrium conditions in LB and suggests that it could act as an RNA sponge to counteract RoxS activity by titrating it away from its targets.

### Global effect of a RosA deletion on the proteome

To determine the global effect of RosA on RoxS and FsrA targets, and potentially identify other roles for this non-coding RNA, we performed a global proteomics analysis comparing the WT and *ΔrosA* deletion strains grown to mid-exponential phase in LB. The proteomes were analysed by label free quantitative proteomics. We detected 1463 proteins in the LC–MS/MS analysis and identified 19 proteins that showed statistically significant (*P* value < 0.05) reduced levels in the *ΔrosA* strain compared to WT (Table 1). Interestingly, seven of these proteins have already been assigned to the FsrA (CitB and SdhA) and RoxS regulons (PpnKB, CitZ, EtfA, SucC and SucD) (14,15,21). The mRNAs encoding most of the other proteins showing reduced levels in the *ΔrosA* mutant were predicted by CopraRNA or IntaRNA (41,42,51) to be direct targets of FsrA and/or RoxS, and have been shown to bind similar metal ions (for example Fe^2+^) and cofactors to the proteins encoded by other RoxS/FsrA mRNA targets. This fits with the general agreement that members of the FsrA and RoxS regulons are involved in regulating genes involved in iron homeostasis and oxidoreduction (14,21). The reduced levels of the FsrA and RoxS targets in the *ΔrosA* strain supports the idea that RosA counteracts regulation by both RoxS and FsrA by acting as a sponge for these two sRNAs.

**Table 1.** Proteomics analysis of the *ΔrosA* strain compared to WT shows reduced levels of RoxS and FsrA targets

Protein	BSU Number	log_2_(ΔS345/WT)	Copra/IntaRNA predicted target	Dysregulated in ΔFsrA or ΔRoxS	Protein description and Biological Process
AcsA	BSU29680	–2.27	RoxS		Acetyl-CoA synthetase utilization of acetate, fatty acids
SpoVS	BSU16980	–1.57	FsrA		Unknown spore coat assembly, spore core dehydratation
CitB	BSU18000	–1.40	FsrA	ΔFsrA	Aconitase, trigger enzyme TCA cycle
NadK2/ PpnKB/YtdI	BSU29540	–1.14	RoxS	ΔRoxS	ATP-NAD kinase NADP biosynthesis
YrhF	BSU27210	–1.07	RoxS		Unknown
EtfA	BSU28520	–0.95	RoxS and FsrA	ΔRoxS	Electron transfer flavoprotein (alpha subunit) fatty acid degradation,
CitZ	BSU29140	–0.95	RoxS and FsrA	ΔRoxS	Citrate synthase 2 TCA Cycle
OdhB	BSU19360	–0.87	RoxS		TCA Cycle 2-oxoglutarate dehydrogenase complex
OdhA	BSU19370	–0.85	RoxS		TCA Cycle 2-oxoglutarate dehydrogenase (E1 subunit)
SucC	BSU16090	–0.84	RoxS	ΔRoxS	Succinyl-CoA synthetase (beta subunit) TCA Cycle
YvyI/Pmi	BSU35790	–0.78	FsrA and RoxS		Mannose-6-phosphate isomerase mannose utilization
GudB	BSU22960	–0.78			Glutamate dehydrogenase, trigger enzyme glutamate utilization, control of GltC activity
CitA	BSU09440	–0.78	FsrA and RoxS		Minor citrate synthase Unknown
SucD	BSU16100	–0.76		ΔRoxS	Succinyl-CoA synthetase (alpha subunit) TCA Cycle
YpbR/DynA	BSU22030	–0.73	RoxS		Dynamin-like protein fusion of membranes
YkrA	BSU14550	–0.62	RoxS		Unknown
YcsA	BSU04000	–0.62	RoxS		Putative tartrate dehydrogenase Unknown
SdhA	BSU28440	–0.61	FsrA	ΔFsrA	Succinate dehydrogenase (flavoprotein subunit) TCA cycle
YpiB	BSU22580	–0.48	FsrA		Unknown

### Deletion of RosA leads to destabilisation of RoxS targets

**Figure 7.**
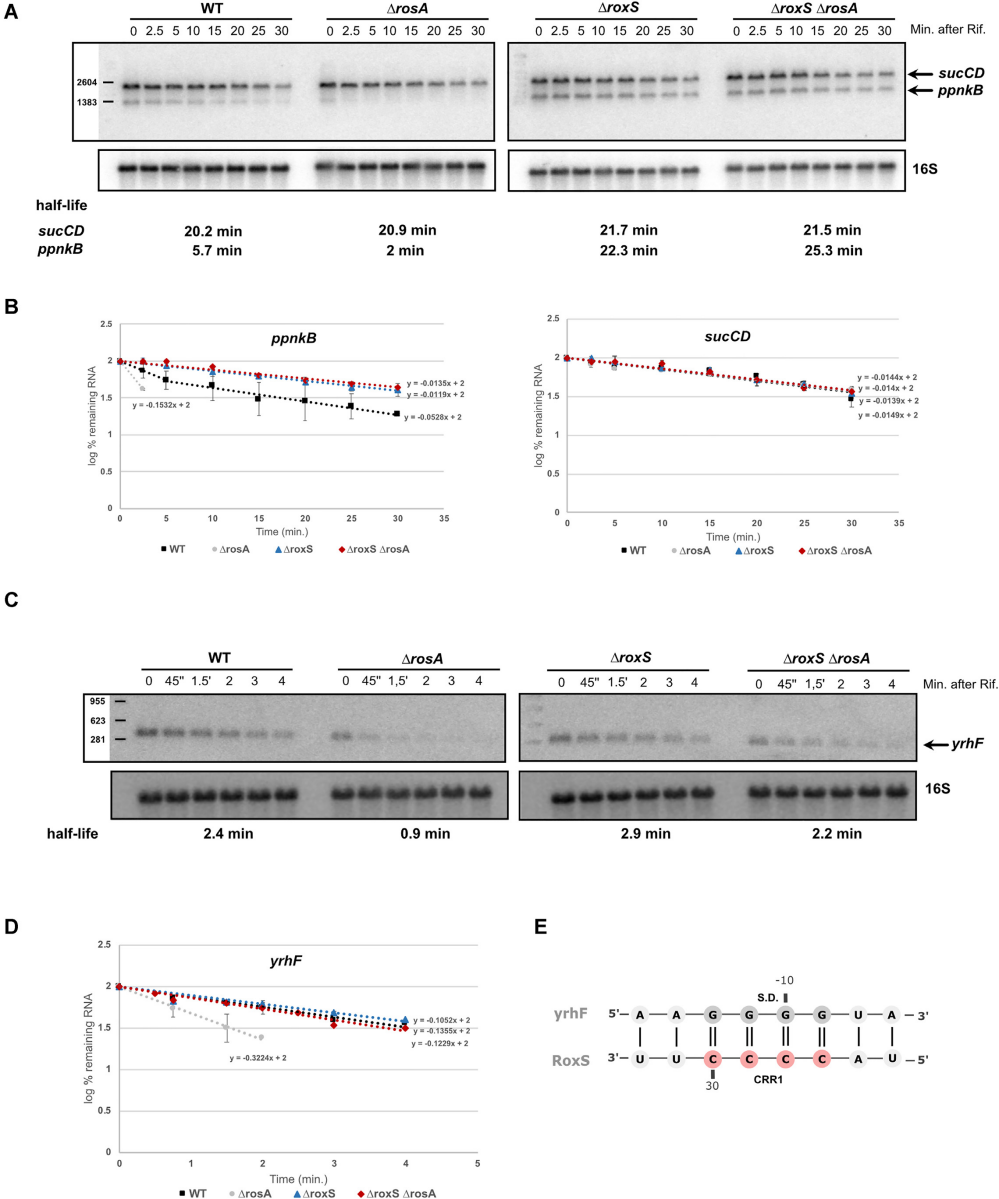
Deletion of RosA alters the turnover rate of RoxS targets. (**A**) Northern blot of total RNA isolated from WT, *ΔrosA*, *ΔroxS* and *ΔrosA ΔroxS* strains probed for the *sucCD* and *ppnKB* mRNA. The blot was re-probed for 16S rRNA as a loading control. Calculated half-lives are shown beneath the autoradiographs. (**B**) Graphs of RNA decay curves showing the log percent remaining RNA for *ppnKB* (left) and *sucCD* (right, in WT, *ΔrosA*, *ΔroxS* and *ΔrosA ΔroxS* strains with their standard deviation calculated from two independent experiments (biological replicates) for each time point after rifampicin addition. For biphasic curves, half-lives were calculated from the initial slope of the curve. (**C**) Northern blot of total RNA isolated from WT, *ΔrosA*, *ΔroxS* and *ΔrosA ΔroxS* strains probed for *yrhF*. The blot was re-probed for 16S rRNA as a loading control. Calculated half-lives are shown beneath the autoradiographs. (**D**) Graphs of RNA decay curves showing the log percent RNA remaining in WT, *ΔrosA*, *ΔroxS* and *ΔrosA ΔroxS* strains with their standard deviation calculated from two independent experiments (biological replicates) for each time point after rifampicin addition. (**E**) Base-pairing prediction between the *yrhF* mRNA and RoxS. The C-rich region of RoxS is coloured in red and the G-rich region of RosA is coloured in grey. The *yrhF* ribosome binding site is indicated (S.D.).

The PpnkB, AcsA and YrhF proteins were among the proteins most affected by the deletion of RosA (Table 1) and are known or predicted to be direct targets of RoxS. *ppnKB* encodes an NAD kinase and was shown previously to be regulated by RoxS through its extensive base-pairing with the *ppnKB* Shine-Dalgarno (SD) sequence (14). RoxS is also predicted to base-pair with the SD sequence of the *acsA* mRNA, encoding acetyl-CoA synthase, and the *yrhF* mRNA, encoding a protein of unknown function (Figures 1A and 7E).

To confirm that effect of RosA deletion on the expression of these proteins is mediated by RoxS, we measured the stability of their corresponding mRNAs in *ΔrosA*, *ΔroxS* and *ΔrosA ΔroxS* mutant strains. If RosA indeed modulates the availability of RoxS to repress its targets, we would predict that the decrease in translation observed in the *ΔrosA* strain due to the additional free RoxS in the cell would lead to a concomitant decrease in the half-lives of these transcripts. In contrast, the effect of the RosA deletion should be abolished when combined with the *ΔroxS* deletion.

In Northern blot experiments performed on cells growing in LB medium, the half-life of the *ppnKB* mRNA was indeed decreased approximately 5-fold in *ΔrosA* cells compared to WT (Figure 7A, B), consistent with the increased amounts of RoxS in the *ΔrosA* strain. As expected, the *ppnKB* mRNA became stable again in the *ΔroxS ΔrosA* double mutant, with a half-life similar to a strain lacking RoxS alone (Figure 7A, B).

As observed for the *ppnKB* mRNA, the deletion of RosA also decreased the stability of *acsA* and *yrhF* mRNA (about >2-fold in both cases) (Figures 1A, B and 7C, D). In contrast, the stability of both mRNAs was similar in *ΔroxS* and *ΔroxS ΔrosA* mutant strains, confirming that the effect of RosA on these mRNA half-lives is RoxS-dependent.

Two other proteins SucC and SucD were also negatively affected by the deletion of RosA (Table 1). These proteins are encoded by the *sucCD* mRNA, previously identified as a RoxS target (14). In LB medium, the rate of degradation of the *sucCD* mRNA was around 20 min in all four strains (WT, *ΔrosA*, *ΔroxS* and *ΔrosA ΔroxS*) (Figure 7A, B). Thus, it was not possible to draw conclusions about the relationship between RosA and RoxS from this experiment. Since we have previously shown that RoxS affects stability of the *sucCD* mRNA in the presence of malate in the medium (17), we tested the impact of RosA on the *sucCD* mRNA in these conditions. To avoid the transcriptional repression of RosA by CcpA in the presence of malate, we used the strain where RosA is under the control of a constitutive promoter, as described above. As anticipated, the constitutive expression of RosA stabilized the *sucCD* mRNA ∼2-fold compared to a strain lacking RosA (Supplementary Figure S10). Moreover, in a strain deleted for RoxS, the *sucCD* mRNA was stable regardless of the presence of RosA.

We confirmed that the effect of the *rosA* deletion mutant was directly related to RosA rather than a polar effect of the mutation on downstream genes by performing complementation experiments. The decreased stability of the *acsA, yrhF, ppnKB* and *sucCD* mRNAs observed in the Δ*rosA* deletion strain in the presence of malate was reversed by the constitutive expression of RosA (Supplementary Figure S10). Furthermore, constitutive expression of RosA no longer had an effect in the *ΔrosA ΔroxS* background, showing that the stabilising effect is dependent on RoxS.

These results confirm that RosA is able to act as a sponge to inhibit RoxS regulation of its targets and provided the basis for the renaming of S345 to RosA, for regulator of sRNA A.

### RosA provides a fitness benefit for *B. subtilis* under conditions of oxidative respiration

**Figure 8.**
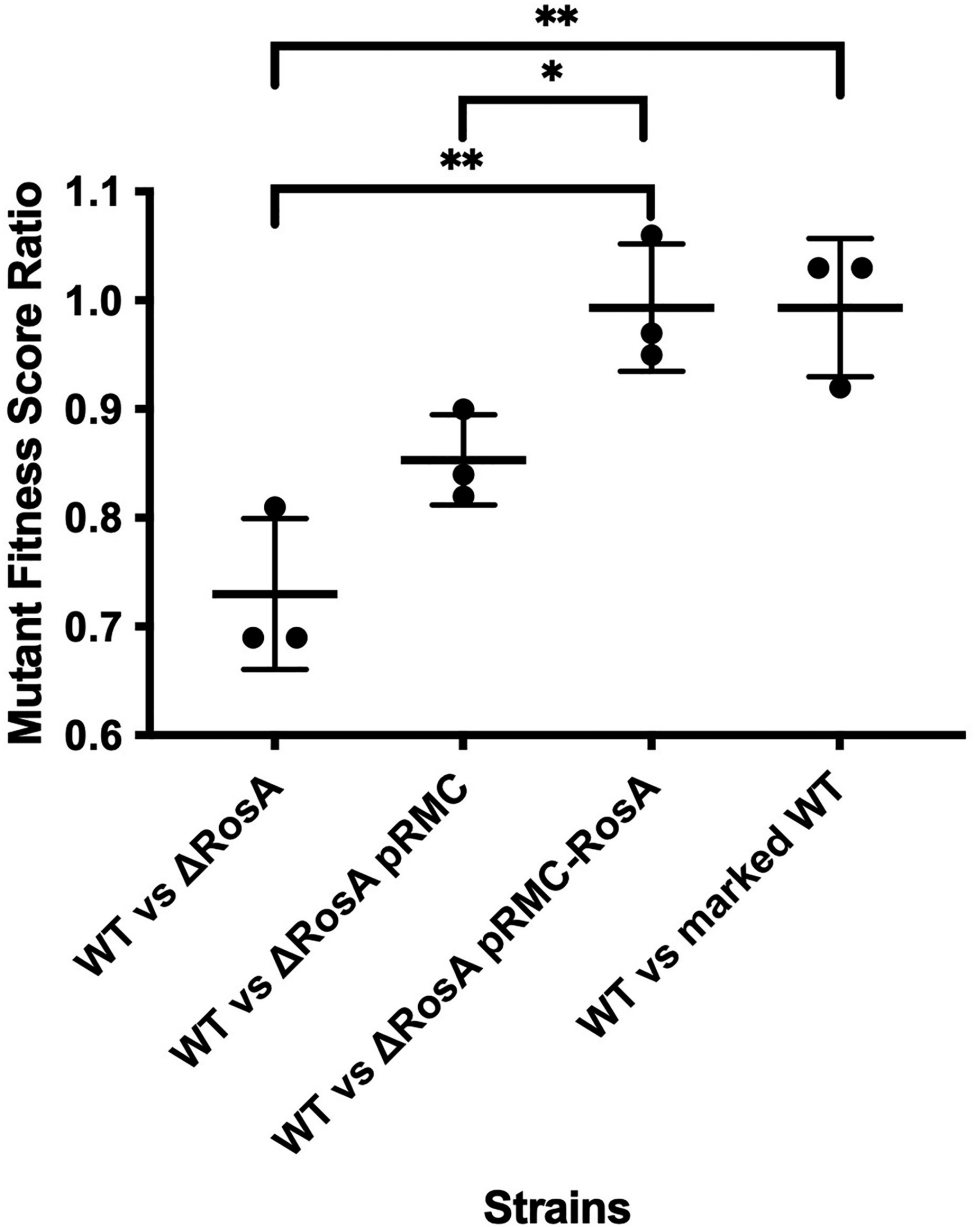
The *ΔrosA* mutant has reduced fitness compared to WT. The fitness deficit of the *ΔrosA* strain in LB was shown by co-culturing *ΔrosA* with the WT strain mixed in a 1:1 ratio. The fitness deficit of *ΔrosA* was restored by cloning the full-size RosA sRNA under the control of its native promoter into the pRMC plasmid. Strains were grown for 24 h and plated on antibiotics to enable CFUs to be determined for each strain in the mixed culture. An antibiotic marked wild type strain was used as a control. Statistically significant differences in fitness between strains calculated using Welch's T test are shown by * and ** (*P value <0.05, ** *P* value < 0.01). The experiment was repeated three times and standard deviations are shown.

Lastly, we asked whether RosA had an impact on global cellular physiology by comparing growth of the *ΔrosA* strain to that of the WT. Since no major difference in growth rate was seen in either LB or in M9 medium, we asked whether there was a more subtle fitness cost to cells lacking RosA by performing competition assays between WT and *ΔrosA* cells in LB medium. We mixed the WT strain marked with a spectinomycin antibiotic resistance cassette and the phleomycin resistant *ΔrosA* mutant at a 1:1 ratio, which was confirmed by colony counts carried out on the starting culture. We then counted the number of *ΔrosA* and WT bacteria after 24 h. The *ΔrosA* strain was recovered at significantly lower levels than the WT suggesting that it is indeed at a competitive disadvantage (Figure 8). In a control experiment, we also competed a phleomycin resistant strain deleted for *yqbR*, a gene located on the Skin prophage region, that was shown to be transcriptionally inactive in LB by Nicolas *et al.* (12). This strain retained a 1:1 ratio with the WT strain after 24 h. We were also able to restore the fitness deficit of the *ΔrosA* strain with ectopic expression of RosA at the *amyE* locus. We propose that the reduction in the levels of enzymes of the TCA cycle, targeted by increased expression of FsrA and RoxS in the *ΔrosA* strain, gives these bacteria a fitness disadvantage, possibly due to their inability to generate ATP as quickly the WT strain.

## DISCUSSION

In this study we report the use of *in vivo* RNA cross-linking using the psoralen AMT to globally identify RNA–RNA interactions occurring in the Gram-positive model organism *B. subtilis*. Our results identified hundreds of potential interactions, including previously well described sRNA–mRNA interactions. Two of three known sRNAs containing C-rich regions in *B. subtilis*, FsrA and RoxS, have been shown to target transcripts encoding essential components of central metabolism using their C-rich regions (14,15,20). In addition to the identification of known targets, we also showed that these two sRNAs interact with a new sRNA, S345, that we renamed RosA in this study.

Deletion of RosA from the *B. subtilis* genome led to an increase of the half-life of RoxS (Figure 6) showing that RosA controls RoxS turnover. In parallel, a proteomic analyses in the *ΔrosA* strain showed reduced levels of known RoxS and FsrA targets, such as the TCA cycle enzymes, SucCD, OdhAB, CitZ, SdhA and CitB, or the NAD kinase, PpnKB (14,15,21). Many of the other proteins with reduced levels in the proteomics experiment were predicted to be targets of either RoxS or FsrA using CopraRNA or IntaRNA (41,51). Two of these proteins are YrhF and AcsA, with the latter encoding a key enzyme in central metabolism that catalyses the conversion of acetate to acetyl-CoA, thus acting as a balancing point for the levels of CoA and acetyl-CoA in the cell (61). Furthermore, the SrtN protein is used by the cell to deacetylate AcsA and this reaction depends on NAD^+^ (62). The goal of RoxS-mediated reduction in AcsA levels may thus be to reduce non-essential NAD^+^ consumption. We confirmed that the stabilities of the *acsA* and *yrhF* mRNAs increased in the absence of RoxS, suggesting that both genes are directly targeted by RoxS. We have also shown that the destabilization effect of the *ΔrosA* mutation on these three RoxS targets (*ppnKB*, *acsA* and *yrhF*) is RoxS-dependant (Figures 1, 7 and Supplementary Figure S10).

In the case of the succinate dehydrogenase (SucCD), the deletion of RosA affected its protein levels but we were unable to measure an impact of either RosA and RoxS on its mRNA stability in LB medium. This suggests that RoxS can affect the translation of *sucCD* mRNA without an effect on its stability in these growth conditions. In the presence of malate, however, RosA stabilized the *sucCD* mRNA in a RoxS-dependant manner (Supplementary Figure S10). These observations suggest that the effects of RoxS on *sucCD* translation and mRNA stability can be uncoupled under specific growth conditions. We have similarly shown in a previous study that RoxS can independently stabilize the *yflS* mRNA and stimulate its translation (17). The benefit of this type of regulation by an sRNA could be its reversibility. Indeed, cells could potentially re-use a repressed mRNA (in this case *sucCD*) for a new round of translation without the requirement of new transcription. This would improve the efficiency of regulation of genes involved in central metabolism that must respond very quickly to changing conditions to adjust energy consumption. Finally, we also showed that the levels of RosA and RoxS are comparable in LB and that one-to-one mixtures of RosA and RoxS *in vitro* result in full-duplex formation. These results show that RosA has the potential to be a highly efficient sponge RNA in *B. subtilis* cells.

In the field of eukaryotic RNA regulation, sponge RNAs are well-accepted as part of the regulatory landscape (63) and this idea has quickly gained traction in bacteria. Indeed, several sponge RNAs have been described in Gram-negative organisms and, intriguingly, many are derived from other transcripts (reviewed in (55–57)). In contrast, RosA is a stand-alone sRNA. Interestingly, another stand-alone sRNA in *S. aureus*, namely RsaI (RsaOG), was also shown to be CcpA-regulated and to interact with the sRNAs RsaG, RsaD and RsaE (the RoxS homologue in *S. aureus*) (64). RsaI, like RosA, contains two G-rich regions to bind to its CRR-containing partners. These results suggest that RsaI and RosA could fulfil the same functions in *S. aureus* and *B. subtilis* and that similar sponge RNA-mediated regulatory pathways exist in Firmicutes to balance the metabolic requirements of the cell. Indeed, RsaI is conserved in the genus *Staphylococcus* but not in Bacilli, while RosA is conserved in some Bacilli but not in the Staphylococci. The role of RsaI as a sponge RNA remains to be definitively proven since the impact of RsaI on RsaE, RsaD and RsaG mRNA targets has not yet been reported. In contrast to RosA, RsaI has also been shown to additionally have a C-rich region used to bind mRNA targets, allowing it to act as both a direct regulator and as a sponge RNA. The absence of equivalent C-rich regions in RosA may limit its regulatory function to that of a sponge RNA, as suggested by our proteomics experiment showing that deletion of RosA only affects the RoxS and FsrA regulons. However, it is interesting to note that RosA potentially encodes a small ORF (sORF) of seven amino-acids, and a ribosome profiling study showed that ribosomes associate with this sORF (65). Moreover, the GRR2 region of RosA, essential for the base-pairing with RoxS, potentially plays the role of the SD sequence for this sORF. This suggests that the sponging of RoxS by RosA could in turn repress the expression of the sORF. Several sORFs encoded by sRNAs have been found in *E. coli* and have additional regulatory functions. Interestingly, one of these proteins, SgrT, is encoded by the regulatory RNA SgrS and is involved in carbon metabolism (66). Further experiments are required to determine whether the RosA sORF has an additional regulatory role.

In this study, we identified four forms of RosA, with different half-lives and none of the main RNases (RNase J1, RNase III, RNase Y and PNPase) could account individually for the processing of RosA to species 2, 3 and 4 (Supplementary Figure S4). The role of RosA in facilitating the processing of RoxS and the possible persistence of a RoxS-RosA duplex in cells (RosA species 3) raises the interesting question of whether RoxS can be recycled from RosA to regulate mRNAs (such as *sucCD*) that prefer the shorter form of RoxS? One could imagine that this duplex might be a reservoir of mostly processed RoxS, that could switch to new partners for which it had a greater affinity. Further experiments are required to explore this possibility.

Expression of RoxS is tightly controlled by two transcription factors, ResD and Rex (14,17). Why then is this additional level of post-transcriptional regulation of RoxS by RosA required? Our previous data suggests that RoxS is involved in readjusting the transitory imbalances in NAD/NADH ratio that occur upon encountering carbon sources such as malate. Through its role in reducing NADH levels, RoxS eventually increases the DNA binding capacity of the transcriptional activator Rex, turning down its own expression. However, RoxS is a relatively stable sRNA, with a half-life of 6 min in a WT strain that increases to >30 min in the absence of RosA. The use of this non-coding sponge RNA is thus likely be a way to dial down RoxS activity more efficiently than by simply turning off transcription, first by neutralizing the C-rich regions involved in the regulation of all known targets so far and then by stimulating its degradation. We propose that RosA accelerates the degradation of RoxS by stimulating the opening of the 5′ stem loop of RoxS encompassing the CRR1 required for the interaction with RosA and the RNase Y cleavage site that produce the truncated form of RoxS, named RoxS (D). In agreement with this hypothesis, the half-life of RoxS observed in the *ΔrosA* strain is similar to that measured previously in a strain deleted for RNase Y (14).

**Figure 9.**
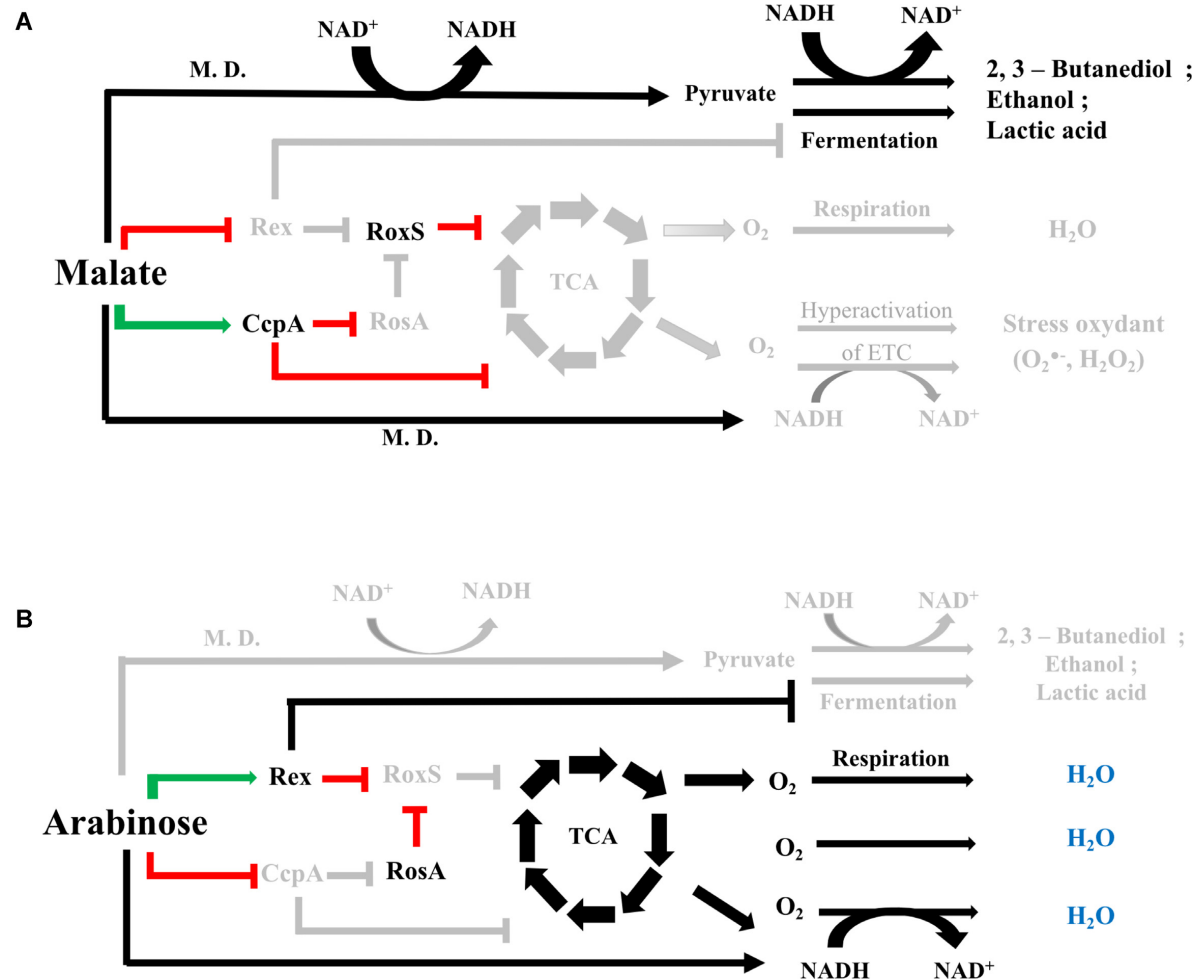
Model for the regulation of the fermentation and respiration pathways by RoxS, RosA and CcpA. (**A**) In the presence of malate as a preferred carbon source, malate dehydrogenases (M.D.) convert malate into pyruvate using NAD as co-factor. The increase in the NADH pool in the cell leads to the inhibition of Rex activity. RoxS is derepressed and regulates its targets (including TCA cycle and respiration enzymes). CcpA is activated and represses expression of numerous genes, including those encoding enzymes of the TCA cycle and the RosA sRNA, thus preventing its sponging effect on RoxS. There could be two goals for this regulation: (1) To avoid the hyperactivation of the electron transport chain (ETC) due to the increase of the NADH pool and limit oxidative stress (2) To activate fermentation pathways by inactivating the Rex repressor, allowing regeneration of NAD^+^. (**B**) In the presence of non-preferred carbon sources such as arabinose, the high NAD^+^/NADH ratio activates Rex, which in turn represses the fermentation pathways and expression of the RoxS sRNA. Carbon catabolite control by CcpA is also inhibited, allowing RosA expression. RosA sponges RoxS sRNA present in the cell and blocks its activity. This cascade of regulation allows the full activation of the TCA cycle and the aerobic respiration in the cell.

What is the metabolic role of this interaction? We determined that RosA is transcriptionally repressed by the main carbon catabolite repressor in *B. subtilis*, CcpA, including during growth in malate known to induce RoxS expression, suggesting that these sRNAs play key roles in reprogramming central metabolism during switches in carbon source. When *B. subtilis* is grown on one of its preferred carbon sources such as malate (67), a large proportion of the carbon is metabolized only as far as pyruvate and acetyl CoA by malate dehydrogenase (Figure 9). These enzymes use NAD as a co-factor, leading to an increase of NADH concentration in the cell, known to inhibit the DNA binding abilities of the transcriptional regulator Rex. This inhibition would allow the transcriptional derepression of RoxS and, instead of directing malate into the TCA cycle, malate would be converted to lactate and acetate *via* fermentation pathways normally repressed by Rex. Fermentation allows the regeneration of NAD+ from NADH. Under these conditions, RosA is repressed by CcpA, which would free RoxS to bind to its targets, including mRNAs encoding enzymes of the TCA cycle which use NAD as co-factor (17). CcpA also represses genes involved in the metabolism of secondary carbon sources and turns down expression many of the enzymes of the TCA cycle and transporters of TCA cycle-intermediates, to ensure resources are not wasted (59,68) (Figure 9). CcpA further activates the transcription of genes whose products are responsible for overflow metabolism when the bacteria are grown on a preferred carbon source. The targeting of these metabolic pathways is strikingly similar to what was observed previously by Durand *et al.* for RoxS, i.e. CcpA and RoxS have many overlapping targets (17), with CcpA acting at the transcriptional level and RoxS acting post-transcriptionally (Figure 9). In contrast, when *B. subtilis* is grown on a non-preferred carbon source like arabinose, the inactivation of the carbon catabolite protein CcpA would allow the transcriptional derepression of the RosA sRNA and other CcpA regulated genes, including those encoding enzymes of the TCA cycle (60). RosA in turn would sponge RoxS and impair the post-transcriptional repression of RoxS targets, also including mRNAs implicated in the TCA cycle. Rex, for its part, represses the fermentation pathways (Figure 9) (69). Thus, the discovery of the RosA RNA sponge under the control of the transcription factor CcpA, provides a missing link between RoxS and CcpA. In other words, RoxS is connected to the CcpA regulon *via* the RosA non-coding RNA, and RoxS ensures an additional, potentially more rapid control at the post-transcriptional level for more than 30% of genes that are regulated by CcpA. The effect of RosA on RoxS also significantly expands the CcpA regulon. These results highlight a complex interplay between transcriptional (Rex, CcpA) and post-transcriptional regulators (RoxS, RosA) in response to carbon sources.

## DATA AVAILIBILITY

RNAseq data has been deposited in ArrayExpress under accession number E-MTAB-8490 and the mass spectrometry proteomics data have been deposited to the ProteomeXchange Consortium via the PRIDE (70) partner repository with the dataset identifier PXD015051.

## Supplementary Material

gkab444_Supplemental_Files

## Notes

Present address: Juan R. Hernandez-Fernaud, Unidad de Investigacion del Hospital Universitario de Canarias. Calle Ofra, s/n. 38320. La Laguna. Canary Islands. Spain.

Present address: Mohammad Tauqeer Alam, Department of Biology, College of Science, United Arab Emirates University, PO Box 15551, Al-Ain, Abu Dhabi, UAE.

Present address: Andrew Millard, Department of Genetics and Genome Biology, University of Leicester, Leicester, UK.

Present address: Emma L. Denham, Department of Biology and Biochemistry, University of Bath, Claverton Down, Bath, UK.
