# Supplementary material for: Identification of an RNA sponge that controls the RoxS riboregulator of central metabolism in Bacillus subtilis
Source: Nucleic Acids Res. 2021 Jun 7;49(11):6399–419. doi: 10.1093/nar/gkab444 (PMC8216469; doi:10.1093/nar/gkab444)
Supplement: gkab444_Supplemental_Files [file gkab444_supplemental_files.zip › Durand et al NAR Supplemental Data Final.pdf]

Supplemental Data

A.

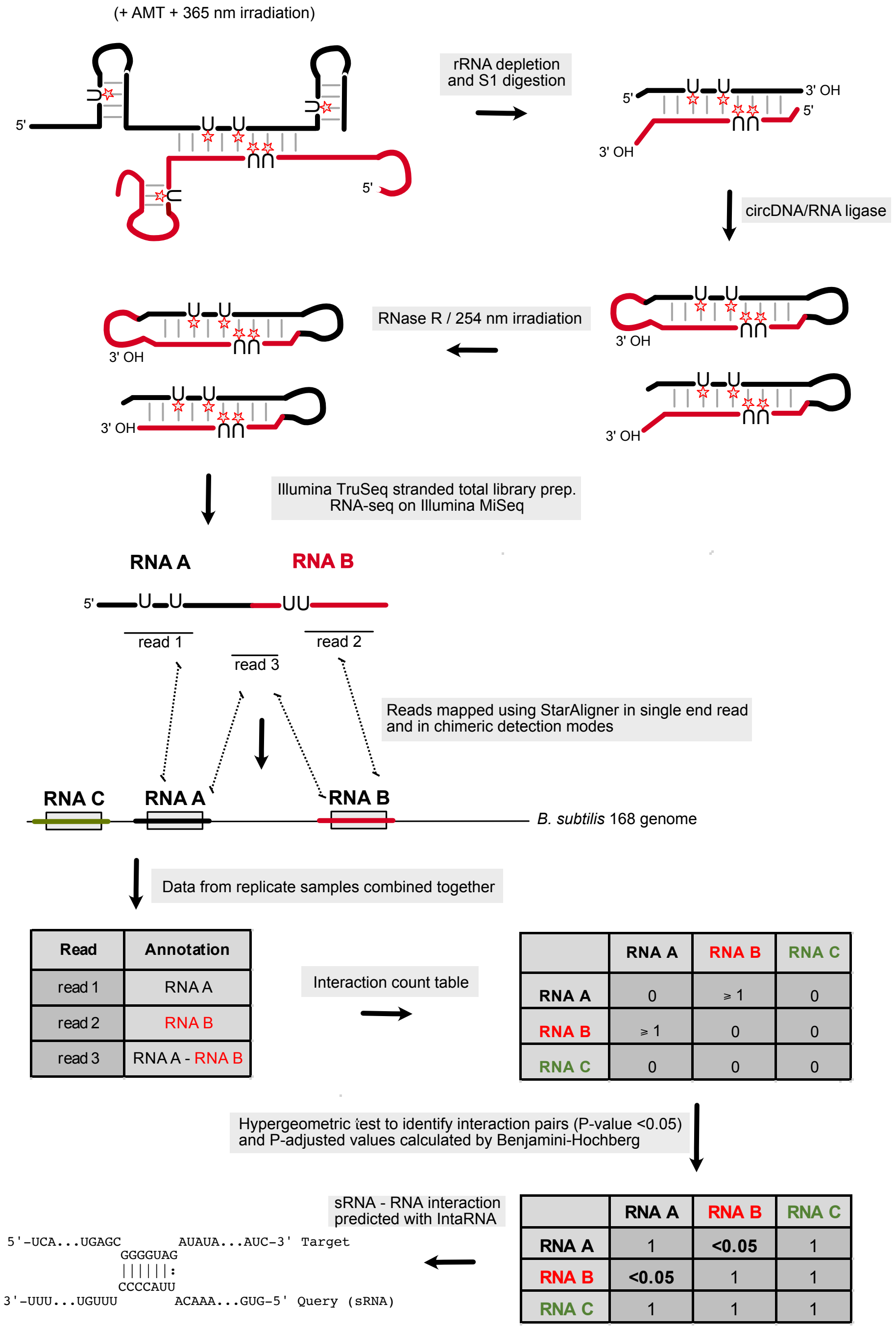

B.

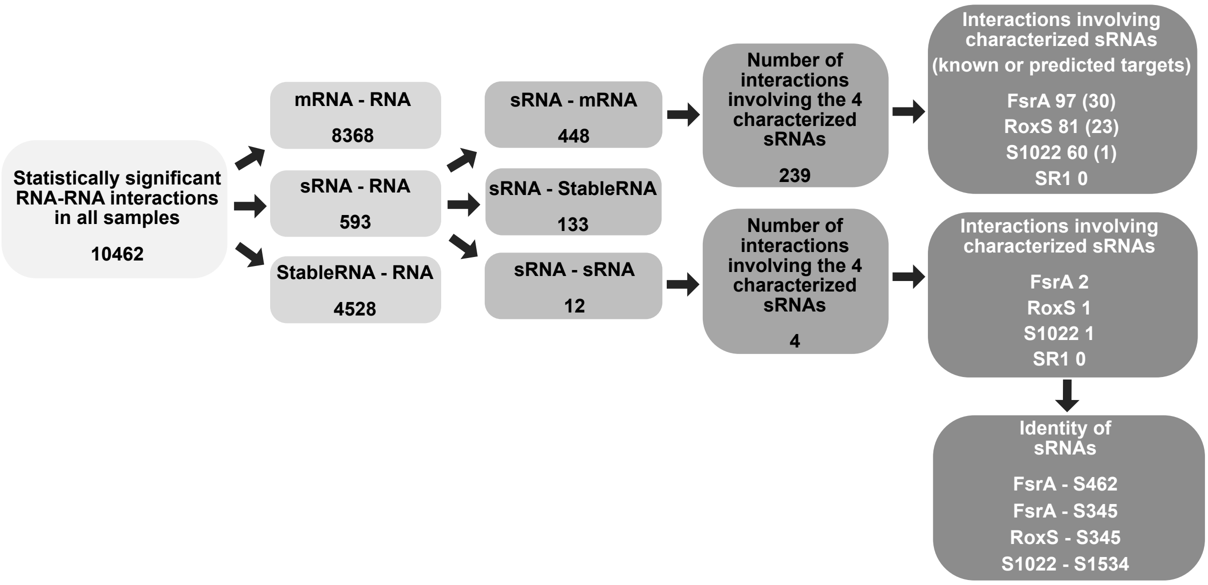

Supplementary Figure 1: Schematic of molecular, bioinformatics and statistical analysis to identify RNA-RNA interactions.

A. The upper part presents the different steps following the cross-linking of RNAs. The middle part shows the bioinformatic analysis performed to map chimeric RNAs. The lower part of the figure details the interaction count matrix used to do the statistical analysis. **B.** Numbers of different RNA interaction types identified in the *B. subtilis* global RNA interaction study**.** RNA interaction types were split into groups based on their classification as mRNA, sRNA or stable RNA. The sRNA interactions were split into the same three groups.

A.

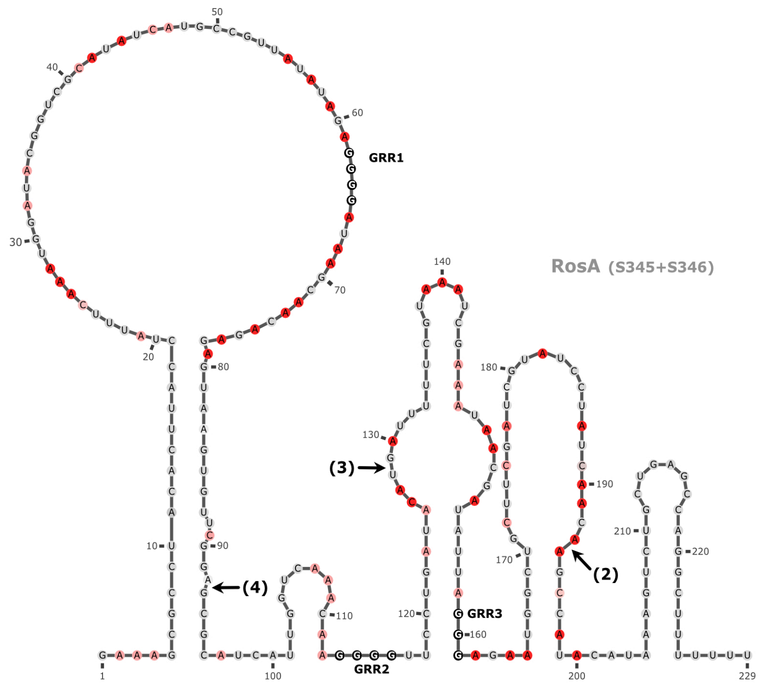

B.

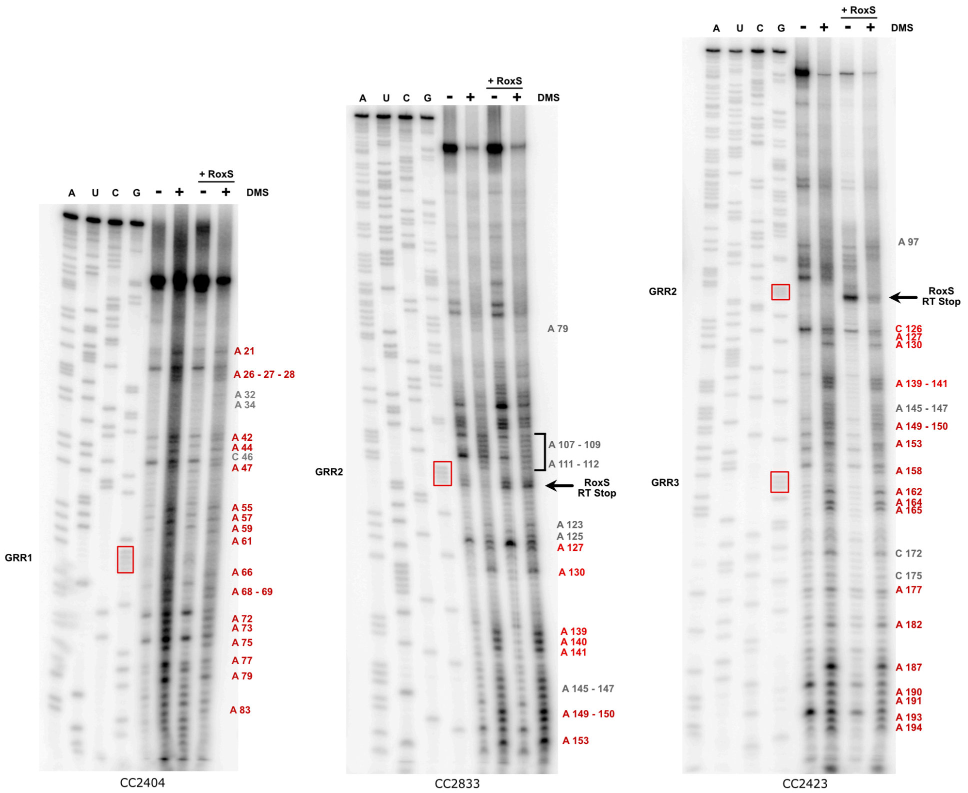

Supplementary Figure 2: Secondary structure analysis of RosA

A. Prediction of the secondary structure of RosA with the RNAfold web server (http://rna.tbi.univie.ac.at/cgi-bin/RNAWebSuite/RNAfold.cgi) and confirmed by DMS probing *in vitro*. G-rich sequences (GRR) are indicated with black circles. Residues reacting with DMS are indicated with red circles (light red: weak reactivity, dark red: strong reactivity). Proposed processing sites (2), (3) and (4) producing RosA species 2, 3 and 4 are indicated by arrows. **B.** Primer extension on RosA RNA treated or untreated with DMS that reacts with unpaired A and C residues. Three different oligos were used for the primer extension assay: CC2404 is complementary to the middle of the RosA sequence overlapping GRR2, CC2833 is 44 nucleotides after GRR2 , and CC2423 is complementary to the sequence close to the transcriptional terminator, corresponding to probes c, d, e in Fig. 3. Reacting residues are indicated to the right of each gel. Residues in grey and red indicate a low and a high reactivity to DMS, respectively. The RoxS-dependent RT stop (arrow) and A-residues with decreased DMS reactivity in the presence of RoxS (square brackets) are indicated in the centre panel.

A.

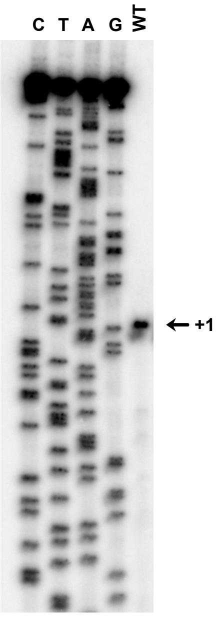

B.

+1

-35 -10

TAAAAAAACCCTTGTCATCGGAAAGGAAAACTGAGATAATAACAT*TGAAAGCGCCT*ACAC

CcpA binding site

TTACCTATTTCAAATGGATACGGTCGCATATCATGCCGTTATATAGAGGGGATAAGCAAC

GRR1

AGAGAGTAAGTGTTCGGAGCGCATCATTGGTCAAACAAGGGGTTCCTGATACATGATTTT

GRR2

TCGTAAATCGAAATAACGATATTAGGGAGAATGGCTGCTTCGATCGTATCCTATCAACAA

GCCATACATAAAGTCTGCTGAGCCAGGCTTTTTTTGCTTT

Supplementary Figure 3: Mapping of the RosA 5' extremity

A. Primer extension to determine the 5'-end of RosA. RNA was extracted from the WT strain grown in LB. The sequencing reaction and the primer extension was carried out with an oligonucleotide localized in the middle of RosA (CC2404). (+1) indicates the 5' end of RosA. **B.** The -10 and -35 sequence of the putative σA promoter are indicated in red. The Cre site for CcpA binding starting at -1 is italicized and underlined. The 3 G-rich regions (GRR) of RosA are underlined. The sequence in blue indicates the terminator of transcription.

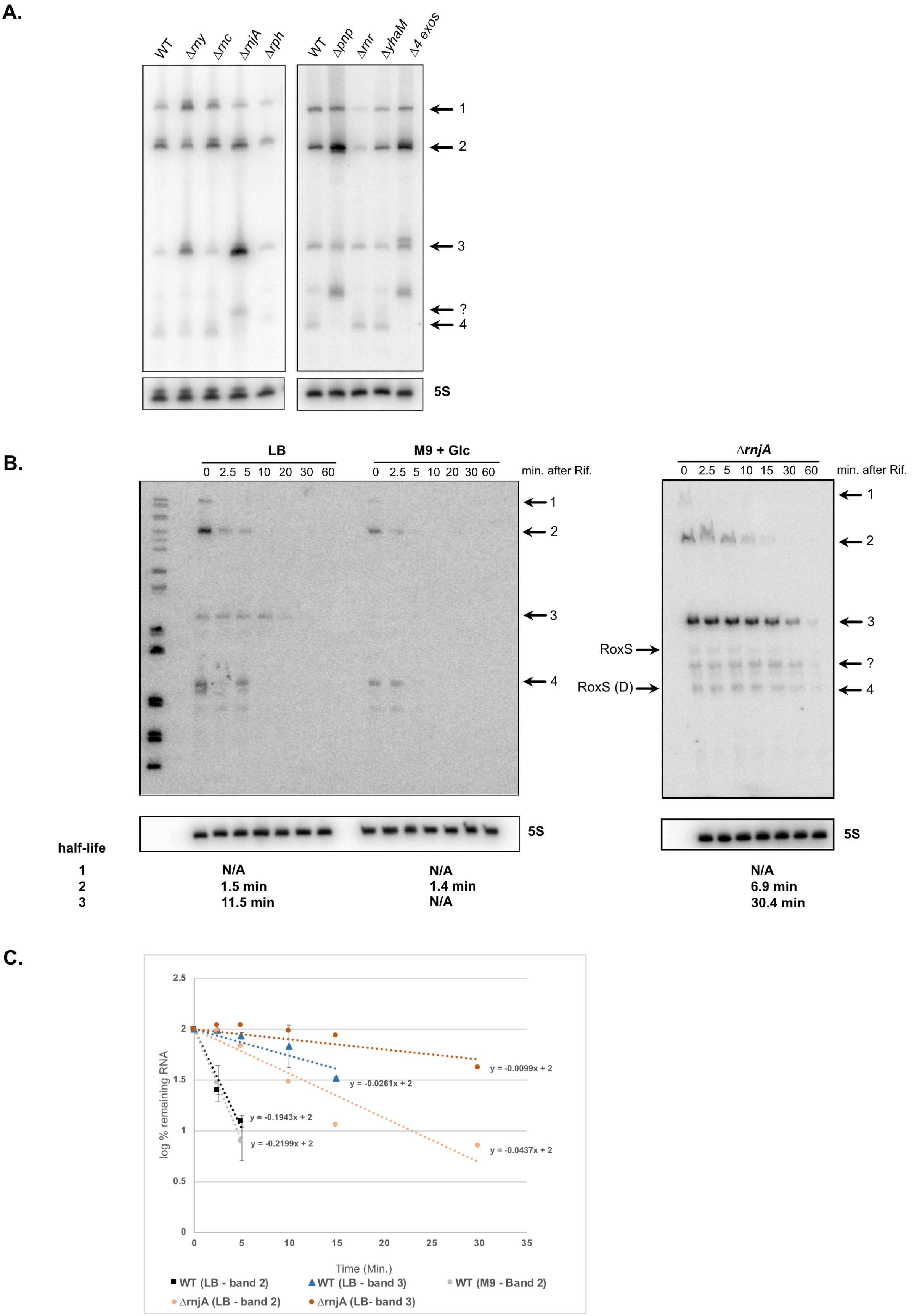

A.
WT
Δrny
Δrnc
ΔrnjA
Δrph
WT
Δpnp
Δrnr
ΔyhaM
Δ4 exos
1
2
3
?
4
5S
B.
LB
M9 + Glc
0 2.5 5 10 20 30 60
0 2.5 5 10 20 30 60
min. after Rif.
1
2
3
4
ΔrnjA
0 2.5 5 10 15 30 60
min. after Rif.
1
2
RoxS
3
?
RoxS (D)
4
5S
5S
half-life
1 N/A N/A N/A
2 1.5 min 1.4 min 6.9 min
3 11.5 min N/A 30.4 min
C.
log % remaining RNA
Time (Min.)
2.5
2
1.5
1
0.5
0
0 5 10 15 20 25 30 35
y = -0.0099x + 2
y = -0.0261x + 2
y = -0.1943x + 2
y = -0.2199x + 2
y = -0.0437x + 2
WT (LB - band 2)
WT (LB - band 3)
WT (M9 - Band 2)
ΔrnjA (LB - band 2)
ΔrnjA (LB- band 3)

Supplementary Figure 4: RosA maturation and degradation in various RNase mutants

A. Northern blot of total RNA isolated from the WT and ribonuclease mutant strains (*Δrny, Δrnc, ΔrnjA, Δrph,* Δ*pnp, Δrnr, ΔyhaM and* Δ4 exos (Δ*pnp Δrnr ΔyhaM Δrph*)) probed for RosA. The four forms of RosA are indicated by arrows. A species whose 5' and 3' boundaries have not been identified accumulates in the Δ*rnjA* strain (indicated by a question mark). **B. Left** Northern blot of total RNA isolated from WT cells grown in LB or M9 + glucose (glc) at times after the addition of rifampicin to inhibit transcription and probed for RosA. **Right** Northern blot of total RNA isolated from the Δ*rnjA* strain at times after the addition of rifampicin to inhibit transcription probed for both RosA and RoxS. Note that band 4 of RosA comigrates with RoxS (D). The blot was re-probed for 5S rRNA as a loading control. Calculated half-lives for species 2 and 3 are shown beneath the autoradiographs, based on graphs shown in panel **C**.

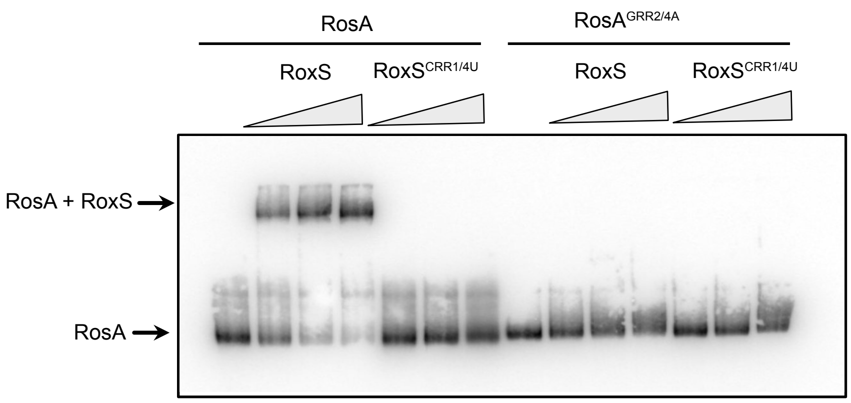

Supplementary Figure 5: EMSA between RosA and RoxS with reciprocal mutations in CRR1 and GRR2

EMSA of WT RosA (FL) or the RosA$^{GRR2/4A}$ mutant (where the four G's were replaced by four A's) with WT RoxS or the RoxS$^{CRR1/4U}$ mutant (where the four C's were replaced by four U's). In each experiment, 5 pmol of RosA was incubated with an increasing concentration (2.5, 5 and 10 pmol) of RoxS, PrsA2 or FsrA RNA, as indicated.

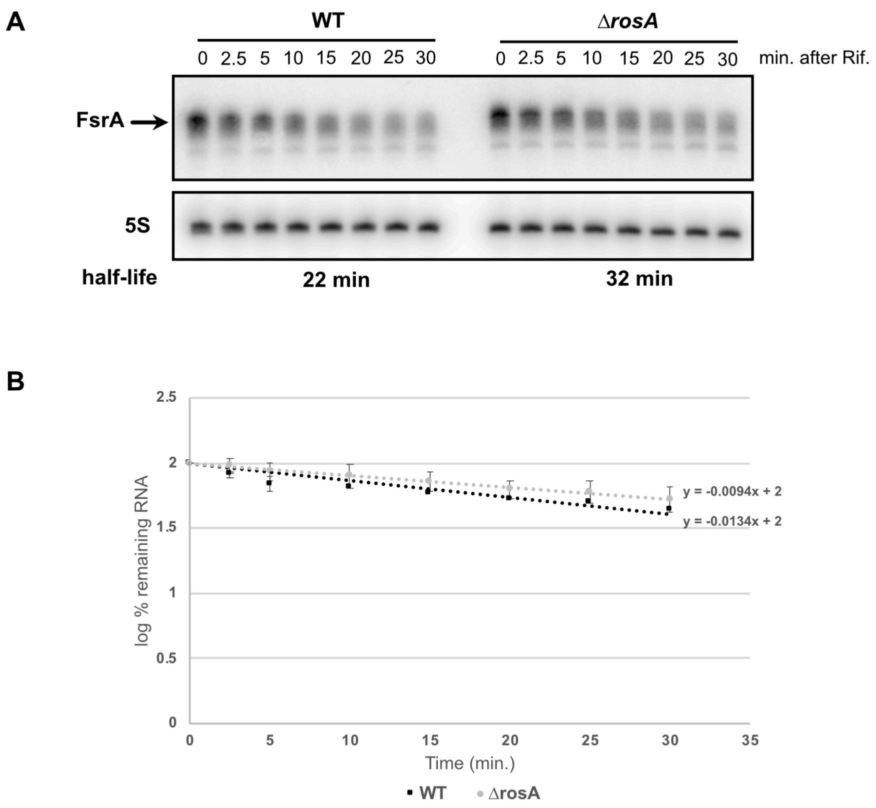

Supplementary Figure 6: Deletion of RosA alters the turnover rate of FsrA

A. Northern blot of total RNA isolated from WT and Δ*rosA* cells grown in LB at times after the addition of rifampicin to inhibit transcription, probed for FsrA. The blot was re-probed for 5S rRNA as a loading control. Calculated half-lives are shown beneath the autoradiographs, based on the graph below. **B.** Graph of FsrA RNA decay curves in WT and Δ*rosA* strains showing the log percent RNA remaining with their standard deviation calculated from two independent experiments (biological replicates) for each time point after rifampicin addition.

A

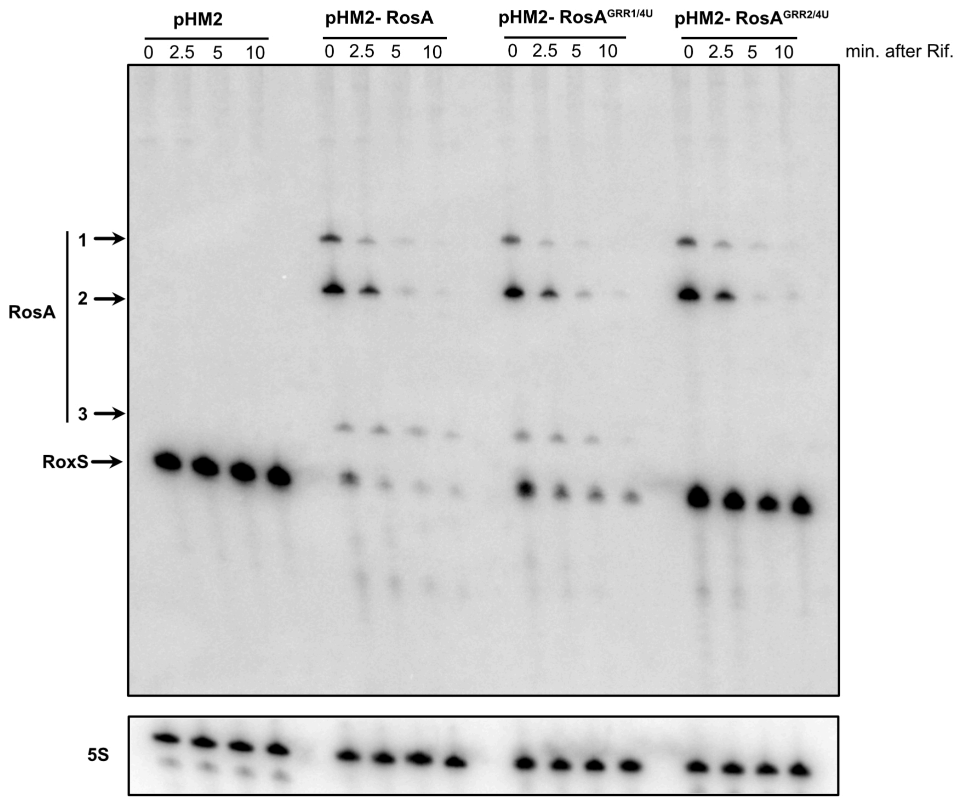

half-life	pHM2	pHM2- RosA	pHM2- RosA$^{GRR1/4U}$	pHM2- RosA$^{GRR2/4U}$
2	N/A	1.3 min	1.6 min	1.4 min
3	N/A	5.7 min	4.9 min	N/A
RoxS	>10	2.2 min	2.2 min	>10

B

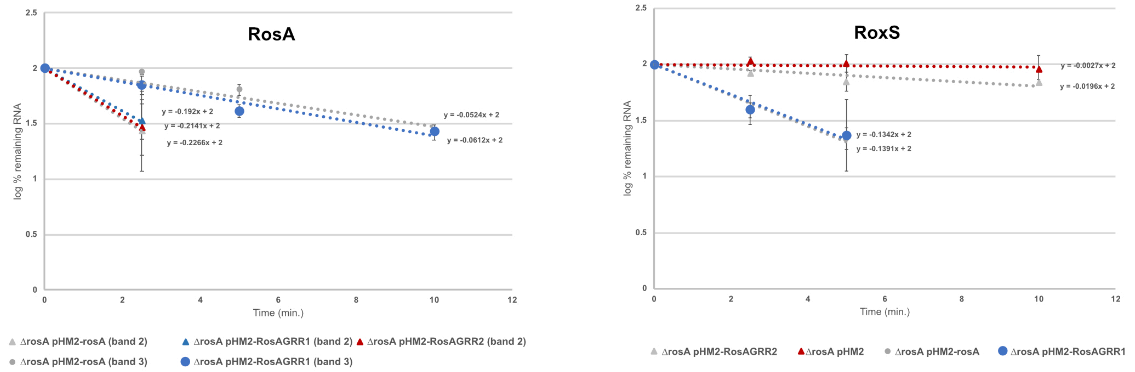

Supplementary Figure 7: Destabilization of RoxS by RosA and RosA mutants.

A. Northern blot of total RNA isolated from strains containing pHM2, pHM2 RosA, pHM2-RosA$^{GRR1/4U}$ and pHM2-RosA$^{GRR2/4U}$ before and after the addition of rifampicin to inhibit transcription, probed for both RosA and RoxS. RosA is under the control of a constitutive

promoter. Bands 1, 2 and 3 refer to the three principal forms of RosA. The membrane was reprobed for 5S rRNA was probed as a loading control (the band just below the 5S corresponds to a remaining RoxS signal). Calculated half-lives are shown beneath the autoradiographs, based on the graph below. **B.** Graph of RosA (left panel, species 2 and 3) and RoxS (right panel) RNA decay curves in a Δ*rosA* strains overexpressing RosA (pHM2-RosA) or RosA mutated in GRR1 or GRR2 (pHM2-RosA^{GRR1} and pHM2-RosA^{GRR2}) showing the log percent RNA remaining with their standard deviation calculated from two independent experiments (biological replicates) for each time point after rifampicin addition.

A

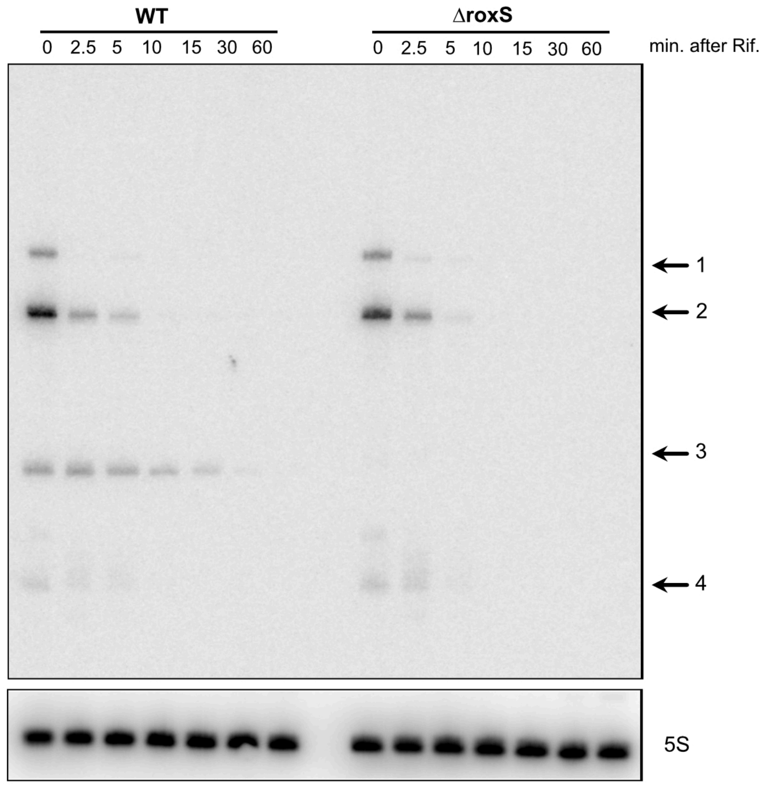

half-life (min)		
2	1.5 min	1.4 min
3	9.3 min	N/A

B

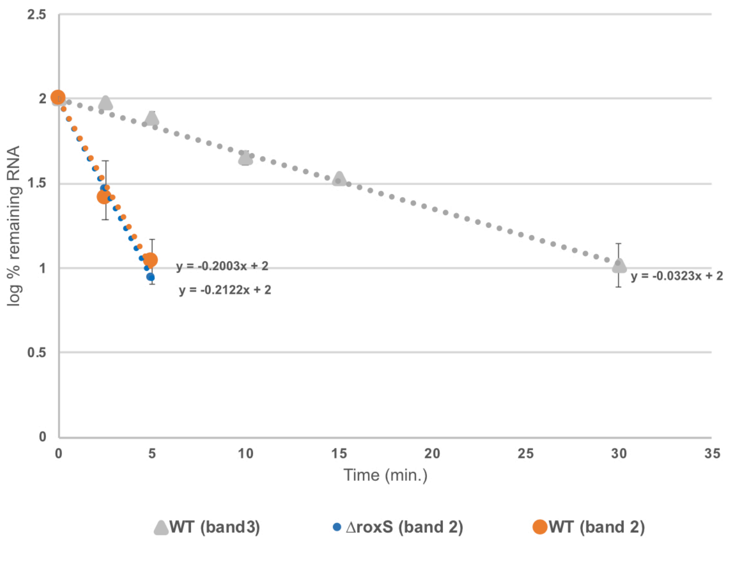

Supplementary Figure 8: RosA processing in a ∆*roxS* mutant strain

A. Northern blot of total RNA isolated from the WT and *ΔroxS* mutant strain before and after the addition of rifampicin to inhibit transcription and probed for RosA. Calculated half-lives are shown beneath the autoradiographs, based on the graph below. **B.** Graph of RosA RNA (species 2 and 3) decay curves in a WT and ∆*roxS* strains showing the log percent RNA remaining with their standard deviation calculated from two independent experiments (biological replicates) for each time point after rifampicin addition.

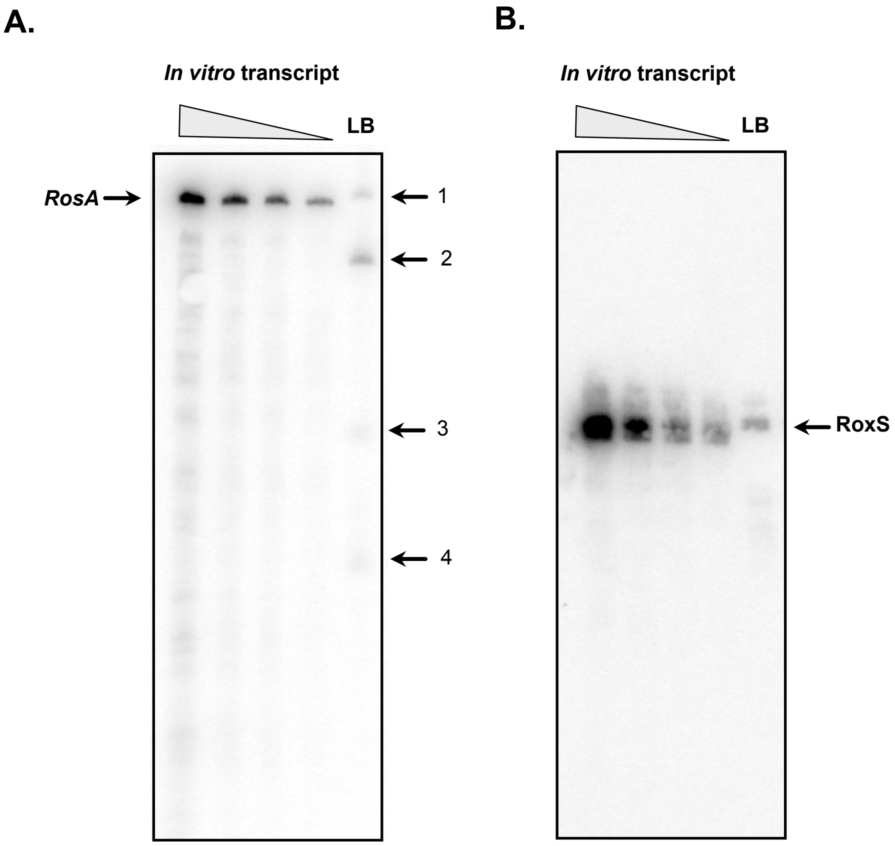

RosA (species 1,2,3 and 4) :

9.5 fmol

RoxS (FL):

9.4 fmol

Supplementary Figure 9: RosA and RoxS are present at similar levels *in vivo*

Northern blot with a defined amount of *in vitro* transcript of (**A.**) RosA (50, 25, 12,5 and 6 fmol) or (**B.**) RoxS (250, 100, 50 and 25 fmol) and 5 µg of total RNA extracted from a WT strain grown in LB (O.D.$_{600\ nm}$=0.6). All the signals detectable for RosA and RoxS in the total RNA lane was taken into account for the quantification. The experiment was repeated 2 times.

A.

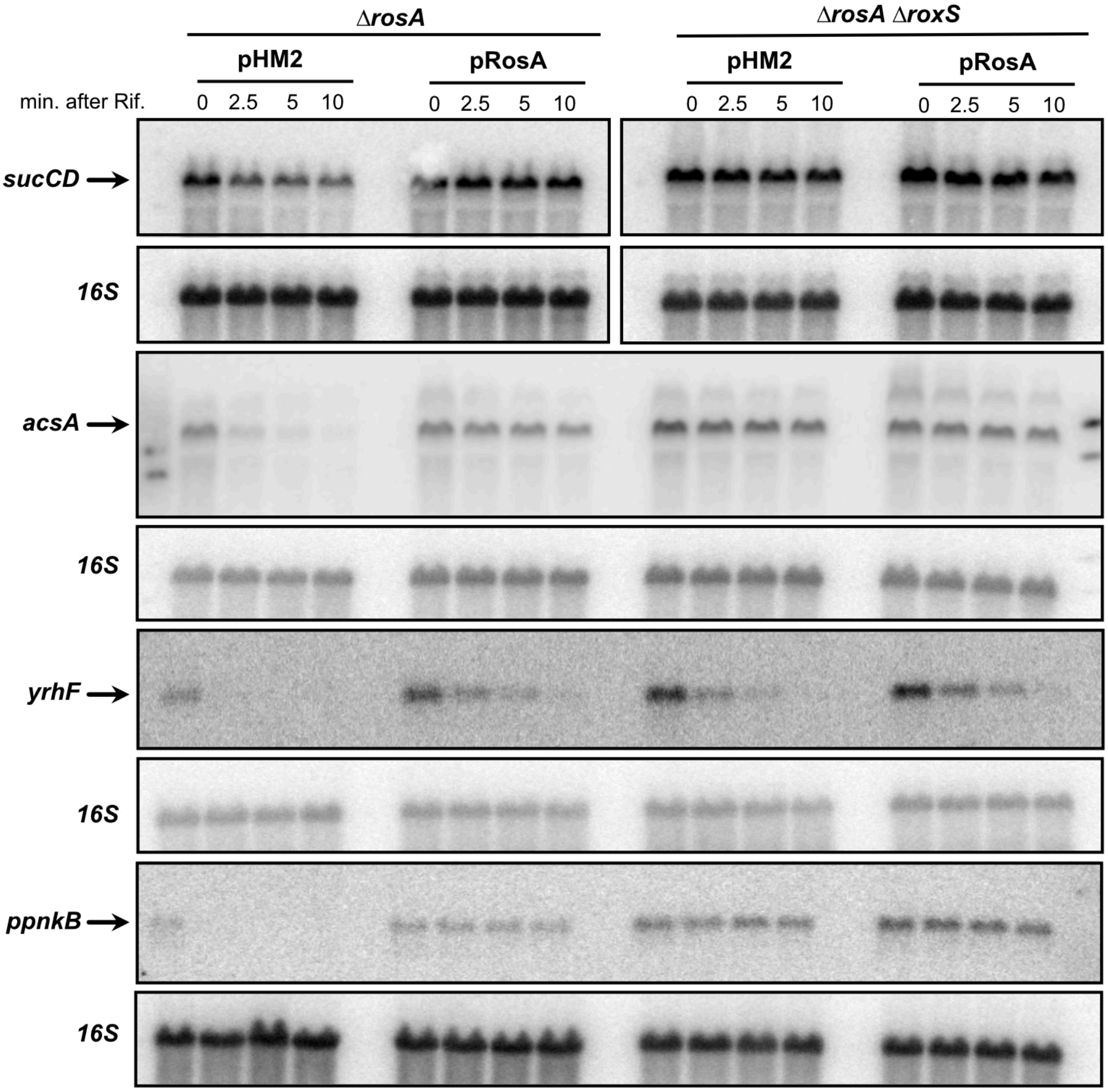

half-life				
sucCD	5.6 min	> 10 min	> 10 min	> 10 min
acsA	3 min	17.8 min	18.9 min	17 min
yrhF	N/A	1.7 min	1.6 min	2 min
ppnkB	N/A	> 10 min	> 10 min	> 10 min

B.

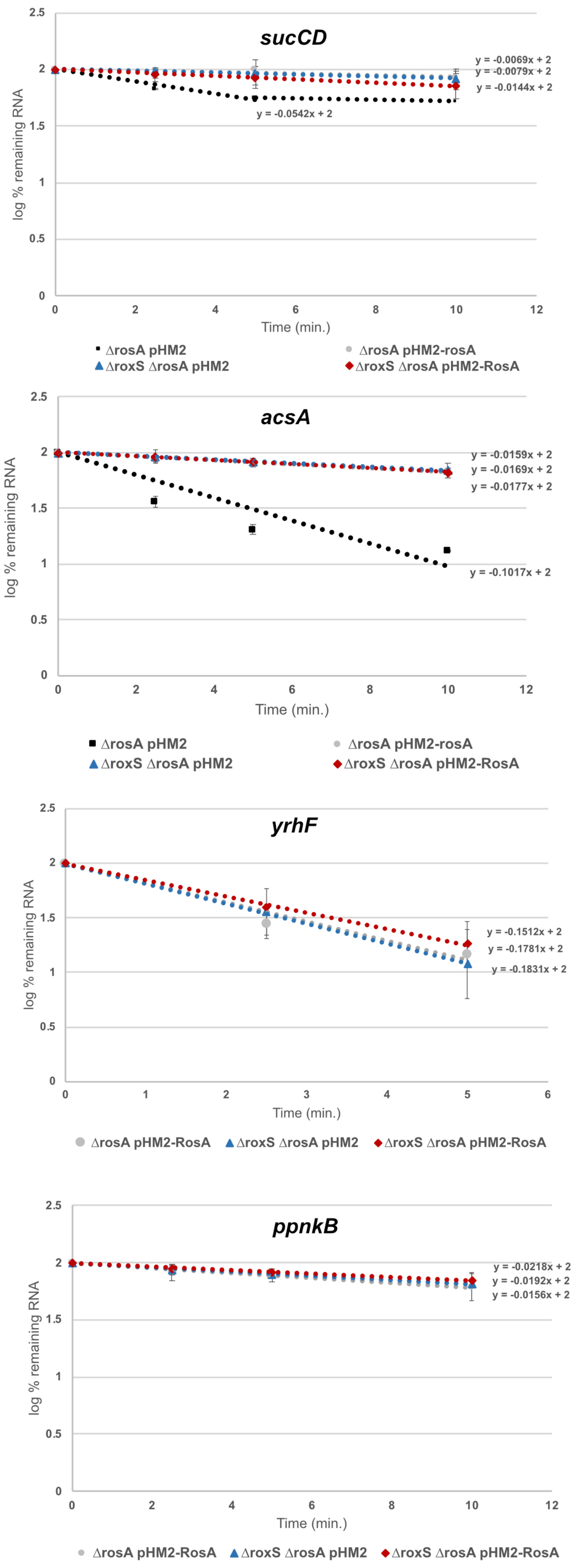
sucCD
y = -0.0069x + 2
y = -0.0079x + 2
y = -0.0144x + 2
y = -0.0542x + 2
log % remaining RNA
Time (min.)
ΔrosA pHM2
ΔrosA pHM2-rosA
ΔroxS ΔrosA pHM2
ΔroxS ΔrosA pHM2-RosA
acsA
y = -0.0159x + 2
y = -0.0169x + 2
y = -0.0177x + 2
y = -0.1017x + 2
log % remaining RNA
Time (min.)
ΔrosA pHM2
ΔrosA pHM2-rosA
ΔroxS ΔrosA pHM2
ΔroxS ΔrosA pHM2-RosA
yrhF
y = -0.1512x + 2
y = -0.1781x + 2
y = -0.1831x + 2
log % remaining RNA
Time (min.)
ΔrosA pHM2-RosA
ΔroxS ΔrosA pHM2
ΔroxS ΔrosA pHM2-RosA
ppnkB
y = -0.0218x + 2
y = -0.0192x + 2
y = -0.0156x + 2
log % remaining RNA
Time (min.)
ΔrosA pHM2-RosA
ΔroxS ΔrosA pHM2
ΔroxS ΔrosA pHM2-RosA

Supplementary Figure 10: *sucCD* mRNA levels in a strain constitutively expressing RosA

A. Northern blot of total RNA isolated from ∆*rosA* and ∆*rosA* *ΔroxS* mutant strains which constitutively express RosA at the *amyE* locus (pRosA), at times after the addition of rifampicin to inhibit transcription, probed for *sucD, ppnkB, acsA and yrhF*. Strains were grown in 2XTY with malate. 16S rRNA was probed as a loading control. **B.** Graph of *sucCD*, *acsA*, *yrhF* and *ppnkB* RNA decay curves in a ∆*rosA* and ∆*rosA* ∆*roxS* strains overexpressing RosA (pRosA) showing the log percent RNA remaining with their standard deviation calculated from two independent experiments (biological replicates) for each time point after rifampicin addition.

Supplementary Tables 1 and 2

Strains and Oligos used in this study

Supplementary Table 3

All statistically significant interactions between FsrA (A) and RoxS (B) across the pairs of sequenced samples. Columns - Target id (BSU number, Nicolas et al locus id, UA id), Target gene name (interacting feature name), start, end, direction, M9 exponential p value, M9 exponential p-adj value, M9 transition p value, M9 transition p-adj value, LB exponential p value, LB exponential p-adj value , LB exponential ∆*fur* p value, LB exponential ∆*fur* p-adj value, min p value, min p value sample, min p-adj value, min p-adj value sample, known target or predicted target (published or predicted by CopraRNA, IntaRNA or TargetRNA), Reference, sRNA interaction bp (region of sRNA predicted by IntaRNA involved in interaction), target interaction bp (start codon = 1), target length (length of the RNA involved in the interaction (where 50 is stated in the final column 50 bp have been added upstream and downstream to account for the transcriptional start site and stop site where a UTR is not known for the RNA), interaction energy (as calculated by IntaRNA), Regions included in target prediction 50 = 50 bp upstream and 50 bp downstream of start codon, 0 = no addition of nucleotides to prediction, utr+cds = utr and cds where the two are known.
